# Design Space Toolbox V2: Automated Software Enabling a Novel Phenotype-Centric Modeling Strategy for Natural and Synthetic Biological Systems

**DOI:** 10.3389/fgene.2016.00118

**Published:** 2016-07-12

**Authors:** Jason G. Lomnitz, Michael A. Savageau

**Affiliations:** ^1^Department of Biomedical Engineering, University of California, DavisDavis, CA, USA; ^2^Department of Microbiology and Molecular Genetics, University of California, DavisDavis, CA, USA

**Keywords:** biochemical systems theory, gene regulatory circuits, System Design Space, synthetic biology, code:python

## Abstract

Mathematical models of biochemical systems provide a means to elucidate the link between the genotype, environment, and phenotype. A subclass of mathematical models, known as mechanistic models, quantitatively describe the complex non-linear mechanisms that capture the intricate interactions between biochemical components. However, the study of mechanistic models is challenging because most are analytically intractable and involve large numbers of system parameters. Conventional methods to analyze them rely on local analyses about a nominal parameter set and they do not reveal the vast majority of potential phenotypes possible for a given system design. We have recently developed a new modeling approach that does not require estimated values for the parameters initially and inverts the typical steps of the conventional modeling strategy. Instead, this approach relies on architectural features of the model to identify the phenotypic repertoire and then predict values for the parameters that yield specific instances of the system that realize desired phenotypic characteristics. Here, we present a collection of software tools, the Design Space Toolbox V2 based on the System Design Space method, that automates (1) enumeration of the repertoire of model phenotypes, (2) prediction of values for the parameters for any model phenotype, and (3) analysis of model phenotypes through analytical and numerical methods. The result is an enabling technology that facilitates this radically new, phenotype-centric, modeling approach. We illustrate the power of these new tools by applying them to a synthetic gene circuit that can exhibit multi-stability. We then predict values for the system parameters such that the design exhibits 2, 3, and 4 stable steady states. In one example, inspection of the basins of attraction reveals that the circuit can count between three stable states by transient stimulation through one of two input channels: a positive channel that increases the count, and a negative channel that decreases the count. This example shows the power of these new automated methods to rapidly identify behaviors of interest and efficiently predict parameter values for their realization. These tools may be applied to understand complex natural circuitry and to aid in the rational design of synthetic circuits.

## Introduction

One of the current challenges in biology is to understand the mapping between a particular genotype and a particular phenotype in the context of a specific environment. The overarching goal, with important consequences to science, biotechnology, and medicine, is to predict the phenotypes that arise from changes in the genotype, the environment or both. However, phenotypes emerge from complex systems of biochemical components that are inherently non-linear, with intricate interactions that cannot be understood through intuition. Thus, it is no surprise that the “genotype-to-phenotype” problem is commonly regarded as one of the grand challenges in modern biology (Brenner, 2000).

A prominent approach to address this difficult challenge is to formulate mathematical models and analyze them to gain detailed insight into the design principles underlying biochemical mechanisms (Savageau, 2009). However, most mathematical models of biological mechanisms are analytically intractable and therefore their study tends to be uniquely tailored and limited in scope.

The conventional strategy for the analysis of mathematical models of non-linear phenomena typically begins with a combination of experimentally measured values for a subset of system parameters and mathematically estimated values for the (often many) remaining parameters (e.g., Sun et al., 2012). The result is an established set of parameter values that serves as the focus for analyses that provide local information regarding system behavior. Thus, this conventional strategy might be termed a parameter-centric approach. When the number of parameters is small and the data is rich, this approach can be very successful. However, it is most often the case that the available experimental data is limited and the number of system parameters is large. Consequently, this approach has severe limitations when attempting to discover the repertoire of potential phenotypes latent in a particular system design.

We have recently developed a radically new modeling strategy that—unlike the parameter-centric approach—does not depend on specific values for the parameters (Lomnitz and Savageau, 2015). This new, phenotype-centric, approach builds on and extends the System Design Space method (Savageau et al., 2009; Fasani and Savageau, 2010; Lomnitz and Savageau, 2013) by (1) enumerating the repertoire of model phenotypes latent in a particular system design, (2) identifying phenotypes that exhibit characteristics of interest, and (3) predicting parameter values for the realization of a specific instance of the system exhibiting the characteristics of interest (Lomnitz and Savageau, 2015).

Here, we present a collection of software tools, the Design Space Toolbox V2, that automates the most difficult steps of this strategy. These software tools build on a previous iteration, the Design Space Toolbox for Matlab®, that formalized automatic construction of the design space for biochemical systems (Fasani and Savageau, 2010). The new tools we present here automate the deconstruction of a model into qualitatively distinct phenotypes—thereby automatically enumerating the phenotypic repertoire of the system (Lomnitz and Savageau, 2015). These tools improve upon the previous iteration by addressing key bottlenecks and expanding upon its capabilities through new technologies that enable analyses not previously possible.

The most important contributions from these new tools include (1) a complete redesign for improved resource management and parallelization of the algorithms for concurrent analysis of model phenotypes; (2) automation of the analysis of local stability through an expansion of the analytical capabilities of the tools; (3) automation of the prediction of parameter values for phenotypes of the system, and (4) automating the co-localization of cases to determine the simultaneous realization and visualization of ensembles of model phenotypes (Lomnitz and Savageau, 2015).

We illustrate the capabilities of these new tools and the thought process guiding the new modeling approach by means of an example. Although we use a model with specified mechanisms for illustrative purposes, in practice one will undoubtedly have only partial information about the underlying mechanisms and one must fill in the missing information by making hypotheses that need to be tested. In our method one need only postulate the architectural information: the topology, signs, and stoichiometry of the interactions. As we have discussed elsewhere (Lomnitz and Savageau, 2015), these are the features of a model that are most readily obtained by experiment or by means of sampling a small number of integers. The more difficult values to determine are rate constants and binding constants, which our method handles automatically in the process of testing the hypotheses. Our method allows for the efficient testing of alternative models by automatic enumeration of the phenotypic repertoire and prediction of model parameters without numerical estimation or sampling of a high-dimensional parameter space. In a recent application we tested 40 different models (hypotheses) and found only five that were consistent with the experimental data (Lomnitz and Savageau, in review).

Following the detailed illustration of the methods, we apply them to a mechanistic model for a new synthetic gene circuit, proposed here, that can exhibit multi-stability involving up to four steady states. Furthermore, we show that this circuit can alternate between three distinct states in a step-wise fashion through the transient stimulation in one of two input channels—a positive channel that results in forward transitions through the three states and a negative channel that results in reverse transitions through the three states. In this way, we describe a genetic counter that can count between three states that—unlike other genetic counters that can count transiently (e.g., see Friedland et al., 2009)—can retain its count indefinitely.

This example shows the power of these new automated tools to provide insight into the underlying design principles of a system involving complex non-linear interactions that are ubiquitous in biology. We also have shown that these tools are useful for designing novel synthetic gene circuits that may be important for a variety of applications from biotechnology (e.g., Martin et al., 2003) to medicine (e.g., Ro et al., 2006), and for gaining insights into more complex natural circuitry (e.g., Benner and Sismour, 2005; Stricker et al., 2008; Mukherji and van Oudenaarden, 2009; Tigges et al., 2009; Kim and Forger, 2012).

## Background

In this section, we review key concepts and definitions from the System Design Space methodology (Savageau et al., 2009) that deconstructs systems based on differences in phenotypes (Lomnitz and Savageau, 2014). As a vehicle to facilitate presentation of the basic concepts, we apply the System Design Space method to a simple example involving a single gene regulator that is autogenously controlled via a positive feedback loop that exhibits the potential for bistability. In a later section, we build on this simple example to show how our automated tools can be applied to a more complex circuit.

We analyze the system by (1) formulating a mechanistic model of a simple biochemical system; (2) recasting the model into the generic Generalized Mass Action (GMA) form; (3) constructing the design space for the recast GMA-System; (4) enumerating the phenotypic repertoire of the model; and (5) analyzing model phenotypes to identify their phenotypic characteristics.

### Formulating a mechanistic model of a biochemical system

**Figure 1:**
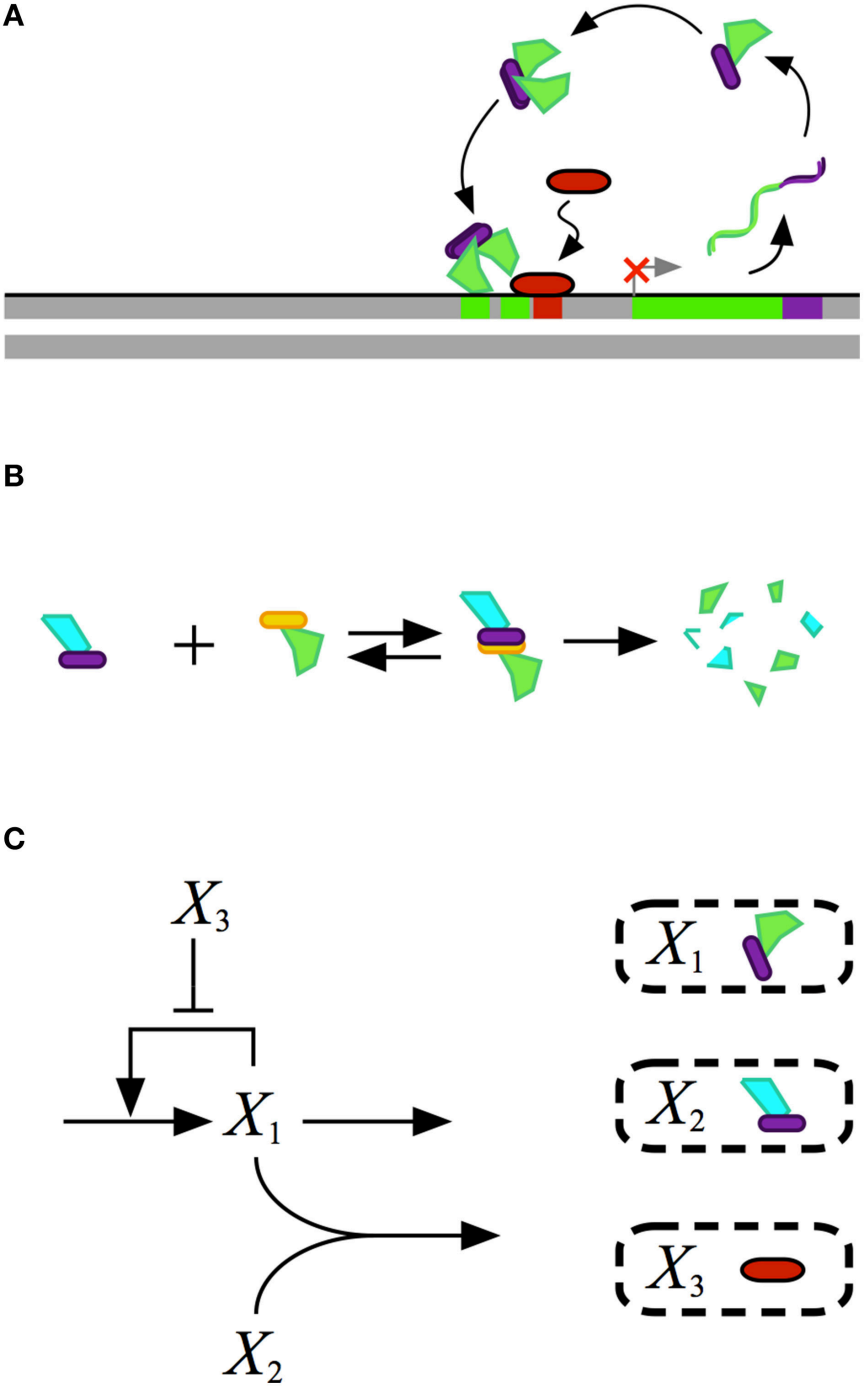
**Conceptual model for the design of a gene regulatory circuit exhibiting positive autogenous regulation**. **(A)** A cartoon of the proposed design showing an autogenously activated gene regulator in green. The regulator is fused with a dimerization domain shown in purple. Homodimerization leads to the active form of the regulator. A repressor, represented by the red capsule, sterically hinders activator binding. **(B)** Binding to a second protein with a complementary dimerization domain leads to a heterodimer that is degraded by cellular proteases or other machinery. **(C)** Abstract representation of the gene circuit design. The activator X_1_, which corresponds to the green protein in the cartoon, autogenously activates its own expression. The bimolecular reaction of X_1_ and X_2_ leads to the heterodimer, which corresponds to the blue-green protein in the cartoon, that is then degraded. The repressor X_3_, which corresponds to the red protein in the cartoon, blocks binding of the activtor to its DNA control region.

Mathematical modeling of biochemical phenomena usually begins with the synthesis of available knowledge from the literature and experimental data that together provide a foundation for generating a particular hypothesis. The hypothesis is usually represented by a conceptual model that contains qualitative information regarding the key components and their interactions, typically visualized using some sort of diagram. An example of a conceptual model for a simple gene regulatory circuit is represented in Figure 1.

Once the qualitative aspects of a system and its interactions have been realized in a conceptual model, we formulate mathematical models by hypothesizing specific biochemical mechanisms involving the elementary rate laws of chemical kinetics and the rational function rate laws of biochemical kinetics (Lomnitz and Savageau, 2013). The result is a system of non-linear differential equations that is analytically intractable in all but the simplest cases (Lomnitz and Savageau, 2014).

In general, the exponents in the power laws that characterize classical chemical kinetics are small integer values, as are the exponents in the rational functions that characterize classical biochemical kinetics. In the case of an enzymatic reaction, the largest exponent in the rate law is equal to the number of reactant binding sites on the enzyme (Wyman, 1964), and this is typically equal to the number of subunits in a multimeric protein (Monod et al., 1965). In the case of a regulator that is a multimeric DNA binding protein, the largest exponent is equal to the number of subunits in the regulator molecule multiplied by the number of specific sites on the DNA to which it binds. Experimental evidence indicates that regulators function as multimeric, typically dimeric, molecules that bind a single recognition site, or possibly a small number of such sites cooperatively, for each transcriptional unit controlled (Mandal et al., 1990; Kim and Little, 1992). If the mechanisms in the model are known, then the exponents will be known; if one has to hypothesize a mechanism, then one has only to sample a small number of fixed integer values for the exponents to characterize the model.

The aspects of a mathematical model that remain fixed for a particular mechanism—independent of the specific values for the parameters that characterize a particular instantiation—are defined as its *architectural features* (Lomnitz and Savageau, 2015). These features include (a) the network topology of interactions, (b) the signs of the interactions, and (c) the number of binding sites involved in the interactions that in turn manifests itself in the exponents found in the power laws of chemical kinetics and in the rational functions of biochemical kinetics, which, as noted above, are fixed integers for a particular mechanism.

A mathematical model for the conceptual system shown in Figure 1 is represented by the following ordinary differential equation (ODE),

(1) $$\begin{array}{r} \frac{dX_{1}}{dt}=\alpha_{1}\left[\frac{1+\rho_{1}{\left(\frac{X_{1}}{K_{1}}\right)}^{2}+\frac{X_{3}}{K_{3}}}{1+{\left(\frac{X_{1}}{K_{1}}\right)}^{2}+\frac{X_{3}}{K_{3}}}\right]-\beta_{1}X_{1}-kX_{1}X_{2} \end{array}$$

where *X*_1_ represents the dependent activator protein; *X*_2_ represents a protein with a complimentary heterodimerization domain, *X*_3_ represents the repressor protein, and their values are treated as independent variables; α_1_ represents the basal level of expression for the synthesis of *X*_1_; β_1_ represents the first-order rate constant for the loss of *X*_1_ by dilution due to exponential growth; ρ_1_ represents the capacity for activation of *X*_1_ synthesis; *K*_1_ represents the concentration of *X*_1_ for the half-maximal rate of synthesis; *K*_3_ represents the concentration of *X*_3_ that results in half-maximal repression; and *k* represents the rate constant for *X*_1_–*X*_2_ heterodimer formation.

This model makes conventional assumptions found in the literature regarding the mechanisms for the control of transcription, and for the translation and loss of stable proteins by dilution due to exponential growth. However, if the mechanisms were unknown, one could postulate alternative mechanisms, as outlined in the Introduction, and test the hypothesized models against experimental data.

### Recasting equations into a generic form

The System Design Space method provides a novel approach to deconstruct mathematical models of biochemical systems (Savageau et al., 2009). At its core, this approach utilizes an innovative definition for model phenotypes that is based on dominant processes that produce sub-systems exhibiting qualitatively-distinct behavior (Savageau et al., 2009).

In order to apply the System Design Space method, the system must first be recast into the canonical GMA form involving a system of differential equations plus algebraic constraints expressed mathematically as,

(2) $$\begin{array}{r} \begin{array}{cc} \frac{dX_{1}}{dt}=\overset{P_{1}}{\underset{k=1}{\sum }}\alpha_{1k}\overset{n+m}{\underset{j=1}{\prod }}X_{j}^{g_{1jk}}-\overset{Q_{1}}{\underset{k=1}{\sum }}\beta_{1k}\overset{n+m}{\underset{j=1}{\prod }}X_{j}^{h_{1jk}}\\ \vdots \\ \frac{dX_{n_{t}}}{dt}= & \overset{P_{n_{t}}}{\underset{k=1}{\sum }}\alpha_{n_{t}k}\overset{n+m}{\underset{j=1}{\prod }}X_{j}^{g_{n_{t}jk}}-\overset{Q_{n_{t}}}{\underset{k=1}{\sum }}\beta_{n_{t}k}\overset{n+m}{\underset{j=1}{\prod }}X_{j}^{h_{n_{t}jk}} \end{array} \end{array}$$

(3) $$\begin{array}{r} \begin{array}{cc} 0=\overset{P_{n_{t}+1}}{\underset{k=1}{\sum }}\alpha_{\left(n_{t}+1\right)k}\overset{n+m}{\underset{j=1}{\prod }}X_{j}^{g_{\left(n_{t}+1\right)jk}}-\overset{Q_{n_{t}+1}}{\underset{k=1}{\sum }}\beta_{\left(n_{t}+1\right)k}\overset{n+m}{\underset{j=1}{\prod }}X_{j}^{h_{\left(n_{t}+1\right)jk}}\\ \vdots \\ 0= & \overset{P_{n}}{\underset{k=1}{\sum }}\alpha_{nk}\overset{n+m}{\underset{j=1}{\prod }}X_{j}^{g_{njk}}-\overset{Q_{n}}{\underset{k=1}{\sum }}\beta_{nk}\overset{n+m}{\underset{j=1}{\prod }}X_{j}^{h_{njk}} \end{array} \end{array}$$

where *n*_*t*_ represents the number of dynamic variables; *n*_*c*_ represents the number of auxiliary variables; *n* = *n*_*t*_ + *n*_*c*_ represents the number of dependent variables; *m* represents the number of independent variables; α_*ik*_ represents the rate constant for the *k*-th positive term of the *i*-th equation; β_*ik*_ represents the rate constant for the *k*-th negative term of the *i*-th equation; *P*_*i*_ and *Q*_*i*_ represent the number of positive and negative terms for the *i*-th equation, respectively; *g*_*ijk*_ and *h*_*ijk*_ represents the kinetic order for the influence of the *j*-th variable on the *k*-th positive and negative term of the *i*-th equation, respectively; *X*_*j*_ represent the *j*-th variable such that the first *n*_*t*_ variables are the dynamic variables, the second *n*_*c*_ are the auxiliary variables and the last *m* variables are the independent variables.

Mechanistic models of biochemical phenomena can be recast exactly into this form by following a well-defined series of steps (Savageau and Voit, 1987). Furthermore, for most biochemical systems the recasting process is straight-forward and involves five simple steps: (1) expanding terms in the numerator by multiplying through by common factors; (2) defining auxiliary variables for each denominator that has multiple terms; (3) rearranging terms in the equation for the auxiliary variables so that the left-hand side is equal to 0; (4) substituting the auxiliary variables for the corresponding denominators; and (5) defining a new system of differential-algebraic equations involving the modified differential equations and the algebraic equations for the auxiliary variables.

We illustrate the process by recasting into the GMA form Equation (1), which involves a typical rational function from biochemical kinetics.

**Step 1**. Expand the numerator of the equation for *X*_1_ by multiplying through by the α parameter.

(4) $$\begin{array}{r} \frac{dX_{1}}{dt}=\frac{\alpha_{1}+\alpha_{1}\rho_{1}{\left(\frac{X_{1}}{K_{1}}\right)}^{2}+\alpha_{1}\frac{X_{3}}{K_{3}}}{1+{\left(\frac{X_{1}}{K_{1}}\right)}^{2}+\frac{X_{3}}{K_{3}}}-\beta_{1}X_{1}-kX_{1}X_{2} \end{array}$$

**Step 2**. Define an auxiliary variable, *X*_100_, equal to the expression in the denominator.

(5) $$\begin{array}{r} X_{100}=1+{\left(\frac{X_{1}}{K_{1}}\right)}^{2}+\frac{X_{3}}{K_{3}} \end{array}$$

**Step 3**. Rearrange terms in the new equation so that the left-hand side of the equation is equal to 0.

(6) $$\begin{array}{r} 0=1+{\left(\frac{X_{1}}{K_{1}}\right)}^{2}+\frac{X_{3}}{K_{3}}-X_{100} \end{array}$$

**Step 4**. Substitute the auxiliary variable for the denominator of the equation from Step 1.

(7) $$\begin{array}{rc} \frac{dX_{1}}{dt}= & \alpha_{1}X_{100}^{-1}+\alpha_{1}\rho_{1}X_{1}^{2}K_{1}^{-2}X_{100}^{-1}+\alpha_{1}X_{3}K_{3}^{-1}X_{100}^{-1}\\ -\beta_{1}X_{1}-kX_{1}X_{2} \end{array}$$

**Step 5**. Define a new system by including the algebraic constraint from Step 3.

(8) $$\begin{array}{rc} \frac{dX_{1}}{dt}= & \alpha_{1}X_{100}^{-1}+\alpha_{1}\rho_{1}X_{1}^{2}K_{1}^{-2}X_{100}^{-1}+\alpha_{1}X_{3}K_{3}^{-1}X_{100}^{-1}\\ -\beta_{1}X_{1}-kX_{1}X_{2} \end{array}$$

(9) $$\begin{array}{rc} 0= & 1+X_{1}^{2}K_{1}^{-2}+X_{3}K_{3}^{-1}-X_{100} \end{array}$$

The result is a differential-algebraic system in a generic form consisting of linear combinations of non-linear terms having a very specific structure (products of power laws) that is capable of representing a broad range of non-linear systems (Lomnitz and Savageau, 2013). It should be noted that from a mathematical perspective both independent variables and system parameters are treated equally within the context of the System Design Space method (Fasani and Savageau, 2010); thus, in this article we use the terms independent variables and parameters interchangeably to refer to their combined set.

### Mathematical definition of qualitatively-distinct phenotypes

The system of equations in the GMA form can be analyzed by using the novel System Design Space method. This method deconstructs complex non-linear systems into a finite number of qualitatively-distinct, non-linear, sub-systems (S-Systems). The qualitatively-distinct phenotypes are mathematically defined in terms of these sub-system equations (Savageau et al., 2009; Lomnitz and Savageau, 2013) and their system behavior is tractable for a variety of system properties (Savageau, 2009; Voit, 2013).

#### Grouping of terms

The mathematical definition of qualitatively-distinct phenotype originates from the structure of the GMA-system. Inspection of this generalized form, shown in Equations (2) and (3), reveals a regular structure: for any *i*-th equation, the right-hand side is a sum of *P*_*i*_ positive terms and *Q*_*i*_ negative terms. Therefore, a system will have a *system signature* that involves a listing of the number of positive and negative terms, i.e., (*P*_1_*Q*_1_*P*_2_*Q*_2_ …*P*_*n*_*Q*_*n*_) (Savageau et al., 2009; Fasani and Savageau, 2010; Lomnitz and Savageau, 2015).

#### Dominant terms

At any given point in the combined variable and parameter space of the system, where each variable and parameter has a specific value, the magnitude of the terms in each equation can be quantified and the terms with a given sign can be ranked based on their relative magnitude. A *dominant term* is defined as the largest term of a given sign for an equation of the GMA-system; and the dominant terms with positive and negative signs are the *dominant positive term* and the *dominant negative term*, respectively (Savageau et al., 2009).

The dominant terms can be uniquely identified based on the index in their corresponding summations. The combination of indices for dominant terms for all the equations yields a unique *case signature* that involves a listing of indices of dominant positive and dominant negative terms in order, i.e., [*p*_1_*q*_1_*p*_2_*q*_2_ …*p*_*n*_*q*_*n*_] (Savageau et al., 2009; Fasani and Savageau, 2010; Lomnitz and Savageau, 2015), where *p*_*i*_ and *q*_*i*_ are the indices of the dominant positive term and dominant negative term of the *i*-th equation, respectively. Note that the system signature (surrounded by parentheses) is differentiated from the case signatures (surrounded by square brackets).

#### Dominant S-Systems

Any point in the variable plus parameter space has a corresponding combination of dominant terms. Because the possible combinations of dominant terms are finite, with the maximum determined by $\prod_{i}^{n}P_{i}Q_{i}$, this partitions the space into a set of discrete “chunks” that are identifiable by their unique case signature (Savageau et al., 2009; Fasani and Savageau, 2010; Lomnitz and Savageau, 2013). Each discrete chunk has a unique combination of dominant terms and, by retaining only the dominant terms and neglecting the non-dominant terms, we can define a *dominant sub-system* that is characteristic of a particular “chunk.”

The dominant sub-systems, defined by retaining only the dominant terms, have a very special structure. These equations are *S-Systems* that have a single positive term and a single negative term that are products of power laws given by the following equations,

(10) $$\begin{array}{r} \begin{array}{cc} \frac{dX_{1}}{dt}=\alpha_{1p_{1}}\overset{n+m}{\underset{j=1}{\prod }}X_{j}^{g_{1jp_{1}}}-\beta_{1q_{1}}\overset{n+m}{\underset{j=1}{\prod }}X_{j}^{h_{1jq_{1}}}\\ \vdots \\ \frac{dX_{n_{t}}}{dt}= & \alpha_{1p_{n_{t}}}\overset{n+m}{\underset{j=1}{\prod }}X_{j}^{g_{1jp_{n_{t}}}}-\beta_{1q_{n_{t}}}\overset{n+m}{\underset{j=1}{\prod }}X_{j}^{h_{1jq_{n_{t}}}} \end{array} \end{array}$$

(11) $$\begin{array}{l} 0=\alpha_{1p_{\left(n_{t}+1\right)}}\prod_{j\;=\;1}^{n\;+\;m}X_{j}^{g_{1jp_{\left(n_{t}+1\right)}}}-\beta_{1q_{\left(n_{t}+1\right)}}\;\prod_{j\;=\;1}^{n\;+\;m}X_{j}^{h_{1jq_{\left(n_{t}+1\right)}}}\\ \;\vdots \\ 0=\alpha_{1p_{n}}\prod_{j\;=\;1}^{n\;+\;m}X_{j}^{g_{1jp_{n}}}-\beta_{1q_{n}}\prod_{j\;=\;1}^{n\;+\;m}X_{j}^{h_{1jq_{n}}} \end{array}$$

The steady-state equations for S-Systems are non-linear but tractable because they become linear when transformed into logarithmic coordinates (Savageau, 2009; Voit, 2013).

#### Dominance conditions

If we had to sample the full (*n* + *m*)-dimensional space of a system—where *n* is the number of dependent variables plus auxiliary variables and *m* is the number of independent variables plus parameters—to identify the regions associated with each qualitatively-distinct phenotype, the usefulness of this approach would be limited. However, the fact that each term is a product of power laws makes possible more extensive analysis of the conditions that partition the continuous variable and parameter space into discrete regions that define the design space of a system.

Dominance can be expressed mathematically through a series of inequalities. The inequalities for the dominant terms of the *i*-th equation are given by,

(12) $$\begin{array}{r} \alpha_{ip_{i}}\overset{n+m}{\underset{j=1}{\prod }}X_{j}^{g_{ijp_{i}}}>\alpha_{ik}\overset{n+m}{\underset{j=1}{\prod }}X_{j}^{g_{ijk}}\forall k=\left\{1,2,3,\ldots ,P_{i}|k\neq p_{i}\right\} \end{array}$$

(13) $$\begin{array}{r} \beta_{iq_{i}}\overset{n+m}{\underset{j=1}{\prod }}X_{j}^{h_{ijq_{i}}}>\beta_{ik}\overset{n+m}{\underset{j=1}{\prod }}X_{j}^{h_{ijk}}\forall k=\left\{1,2,3,\ldots ,P_{i}|k\neq q_{i}\right\} \end{array}$$

which can be transformed to yield a series of linear inequalities in the logarithm of the variables,

(14) $$\begin{array}{rc} \log\alpha_{ip_{i}}+ & \overset{n+m}{\underset{j=1}{\sum }}g_{ijp_{i}}\log X_{j}>\log\alpha_{ik}+\overset{n+m}{\underset{j=1}{\sum }}g_{ijk}\log X_{j}\\ \forall k=\left\{1,2,3,\ldots ,P_{i}|k\neq p_{i}\right\} \end{array}$$

(15) $$\begin{array}{rc} \log\beta_{iq_{i}}+ & \overset{n+m}{\underset{j=1}{\sum }}h_{ijq_{i}}\log X_{j}>\log\beta_{ik}+\overset{n+m}{\underset{j=1}{\sum }}h_{ijk}\log X_{j}\\ \forall k=\left\{1,2,3,\ldots ,P_{i}|k\neq q_{i}\right\} \end{array}$$

Because these inequalities are linear, they have the following characteristics: (1) each condition defines a half-space of the (*n* + *m*)-dimensional space (i.e., half the (*n* + *m*)-dimensional space); and (2) the intersection of all the half-spaces yields either (a) an (*n* + *m*)-dimensional *dominance polytope* (i.e., there is a feasible region for the phenotype in the state plus parameter space) or (b) a null region (i.e., there is no feasible region for the phenotype anywhere in the combined state plus parameter space). The validity of the dominance polytope can be determined very efficiently and is typically the first phase of a linear programming problem (Vanderbei, 1996).

#### Boundary conditions

The steady-state solution of a dominant S-System is linear in logarithmic coordinates (Savageau, 2009). The boundary conditions for validity of the corresponding phenotype are obtained by substituting the linear solution for the steady state into the linear dominance conditions, to yield boundaries for the dominant sub-system that are linear in logarithmic space (Savageau et al., 2009; Fasani and Savageau, 2010). Each boundary condition defines an m-dimensional half-space and the intersection of these half-spaces yields an *m*-dimensional phenotypic polytope.

From a geometric perspective, the steady-state solution defines an *m*-dimensional *solution hyperplane* that “cuts” through the (*n* + *m*)-dimensional *dominance polytope*. The boundary conditions are the edges at the intersection between the solution hyperplane and the dominance polytope, which yields the phenotypic polytope of the system.

However, the boundary conditions may not necessarily yield a feasible region because of two reasons: (a) the dominant S-System is underdetermined and has no steady-state solution or (b) the solution hyperplane and the dominance polytope do not intersect anywhere in the (*n* + *m*)-dimensional space. The validity of the feasible region can be determined in the same way as the validity of the dominance polytope by using linear programming methods (Fasani and Savageau, 2010).

#### Qualitatively distinct phenotypes

The dominant S-Systems capture the behavior of the system's dominant processes contributing to the synthesis and loss for each species. These non-linear sub-systems, with particular phenotypic characteristics, capture the dominant behaviors of the full system. These sub-systems are valid representations of the system behavior within mathematically defined boundaries that are analytically determined by the system equations themselves. The combination of a characteristic sub-system and mathematically defined boundaries partitions parameter space into a finite number of regions where the system behavior has a series of characteristic traits. The result is a mathematical definition for qualitatively-distinct phenotypes that is based on the processes of a given system that are dominant in a particular context (Savageau et al., 2009; Lomnitz and Savageau, 2015).

#### Phenotypic repertoire

The phenotypic repertoire is defined as the collection of qualitatively-distinct phenotypes (valid phenotypic polytopes), integrated into a space-filling structure known as the system design space (Lomnitz and Savageau, 2015).

## Design Space Toolbox V2

It is widely recognized that the phenotype-to-genotype challenge is difficult in large part because the tools available for the analysis of non-linear systems have little power to explore the global landscape of system behavior. Thus, most analyses rely on estimating values for the parameters and analyzing the system at a local level. The System Design Space method addresses some of these limitations by providing detailed information about the system behavior from a global perspective (Lomnitz and Savageau, 2014). It does this by enumerating the repertoire of a system's qualitatively-distinct phenotypes and identifying a subset of phenotypes of interest. It achieves this by deconstructing intractable non-linear systems into tractable non-linear sub-systems that can be reassembled to define a system's design space (Savageau et al., 2009).

We have recently applied this methodology to a variety of biochemical systems that exhibit rich behaviors including multi-stability (Savageau and Fasani, 2009; Martínez-Antonio et al., 2012; Fasani and Savageau, 2013) and oscillations (Lomnitz and Savageau, 2013, 2014, 2015). Other examples involve natural gene circuits that play crucial roles in the transitions between alternative modes of biological operation [e.g., aerobic to anaerobic growth (Tolla and Savageau, 2010, 2011; Tolla et al., 2015), growth phase transitions (Martínez-Antonio et al., 2012) and phage λ transition between lysogenic and lytic growth (Savageau and Fasani, 2009)]. However, in each of these examples, the construction and analysis of the system's design space was significantly simplified by automating and systematizing the System Design Space method. This was first made possible via a collection of software tools known as the Design Space Toolbox for Matlab® (Fasani and Savageau, 2010).

The Design Space Toolbox for Matlab® provided a series of innovations that systematized the analysis of complex systems: it automated (1) construction of a System Design Space; (2) enumeration of the qualitatively-distinct phenotypes of a given system; and (3) the local analyses of the dominant S-System equations. Through these innovations it has provided insight into the fundamental principles underlying a variety of natural systems (Savageau and Fasani, 2009; Tolla and Savageau, 2011). Although these tools have paved the way for more complicated systems to be analyzed by the System Design Space method, it was clear that the implementation of these tools had severe limitations as it pertains to performance when analyzing larger systems. Here we present a second iteration of software tools, the Design Space Toolbox V2, that redesigns the computational approach, enables more complex circuitry to be analyzed, and extends the possible analyses through additional functionality.

### Technology overview

The original toolbox was built within Matlab® as a collection of .m scripts. There were many advantages that resulted from this decision: The Matlab® environment provided access to a variety of tools for symbolic algebra, linear algebra and linear optimization. Furthermore, it provides a rich scientific programming platform with its own interpreted language for rapid iterations between model formulation and model analysis. Furthermore, it provides fast vectorized operations that performed much better than iterated loops in its own language. These properties of the environment were critical in the design choices for the original toolbox, which improved performance by applying vector operations where possible and by providing an application programming interface that was part of the larger Matlab® ecosystem.

However, with these design choices come several limitations: The Matlab® environment provides access to limited system resources and its use of vectorized operations for faster performance had huge memory requirements that limited feasibility for larger problems.

**Figure 2:**
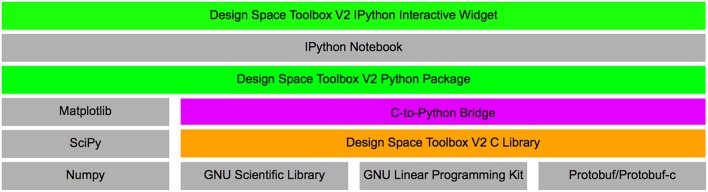
**Overview of the Design Space Toolbox V2**. Components are represented by rectangles, stacks represent component dependencies. Different component types are represented by color: Python packages (green); C library (orange); C/Python wrapper (purple); and third-party dependencies (gray).

Here, we present a novel set of tools using very different design choices. This new collection of tools is comprised of a stand-alone library, written in the C language, that implements its own symbolic algebra engine and leverages open-source compiled libraries for linear algebra (Gough, 2009) and linear optimization (via the GLPK library). This new toolbox applies concurrent approaches to leverage the “embarrassingly parallelizable” nature of the System Design Space approach by analyzing each qualitatively-distinct phenotype of the system independently from every other qualitatively-distinct phenotype using multi-threaded concurrent algorithms. A visual representation of the technology in the Design Space Toolbox V2 is shown in Figure 2.

This new software library applies many of the same concepts and theory of the previous version to automate the construction of a system design space, but involves a complete redesign of the tools for better memory management and parallelization for concurrent analysis. It also extends the original toolbox by providing an extensive library, with over 648 exposed functions, for the analysis of the system and its phenotypes. The new functionality of the toolbox includes: (1) automating the local stability analysis for model phenotypes; (2) enumerating the vertices of the feasible regions in up to three dimensions, both numerically and symbolically; (3) extending the capabilities of the symbolic algebra component to facilitate the analytical discovery of design principles; (4) defining constraints on the dependent variables and parameters of the system (i.e., to define architectural constraints and biological constraints), among many others.

The most important innovation provided by these new software tools is the enabling of a radically new modeling strategy. It does this by facilitating prediction of values for the parameters that can be used to focus computational effort on regions of parameter space that exhibit characteristics of particular interest. It achieves this automatically by (1) enumerating the repertoire of model phenotypes; (2) predicting sets of parameter values for any model phenotype; (3) predicting sets of parameter values for the simultaneous realization and visualization of an ensemble of model phenotypes; and (4) predicting sets of parameter values for the simultaneous realization of an ensemble of model phenotypes that are phased to achieve a specific progression of behaviors (Lomnitz and Savageau, 2015).

Using this approach, we have been able to identify parameter values for a class of systems that display rich behaviors including monostability, bistability, sustained oscillations, and bifurcations among them (Lomnitz and Savageau, 2013, 2014, 2015).

### Analysis via the python package

In this sub-section we illustrate the steps involved in constructing and analyzing a mechanistic model by an application of the toolbox to the simple example given by Equations (8) and (9). These methods will then be applied to a more complicated circuit that exhibits more interested behaviors in Section Example Applied to a Synthetic Memory Module. The simple example and the examples found later in this article illustrate the use of the Python Package within the Design Space Toolbox V2. This level of the toolbox offers access to most of the power of the C library within an interpreted environment similar to Matlab® for rapid scientific programming and prototype analyses.

The Design Space Toolbox V2 also includes a graphical user interface embedded within the IPython Notebook that facilitates its use by new users, with examples readily available online. However, the analyses presented in this Article are not reproducible using the graphical user interface and require the Python Package that has greater access to a wider set of functions.

### Construction of the system design space

The first step in the analysis using the Design Space Toolbox V2 is to prepare the Python environment, which requires importing the python package into the current session:


import dspace


Once the environment has been initialized, the next step is to construct the appropriate computational objects that are used to formulate and analyze the mechanistic model. This entails refactoring the system of equations into a computer-readable format, simply a list of equations using a string representation (^*^ represents multiplication, $\hat{r}$epresents power operator., represents d/dt). The differential equations and algebraic constraints are expressed explicitly by defining both sides of the equations. For example, the system described by Equations (8) and (9) is represented using the following string representation,


string_eq = [‘X1. = a1*X100^-1\
  
+ a1*rho1*X1^2*K1^-2*X100^-1\
  
+ a1*X3*K3^-1*X100^-1\
  
- b1*X1\
  
- k*X1*X2',
  
‘0 = 1 + X1^2*K1^-2 + X3*K3^-1 - X100’
  
]


and then the equations are parsed by the symbolic algebra component to construct an object of the Equations class that represents the system equations including auxiliary variables, which must be defined explicitly:


equations = dspace.Equations(string_eq,
       axuliary_variables=[‘X100’])


At this point, each string is used to construct an object of the Expression class, which parses the string and builds an abstract syntax tree representation that handles symbolic manipulation and evaluation of mathematical expressions within the design space toolbox. The Equations class can then be used to construct an object of the DesignSpace class,


ds = dspace.DesignSpace(string_eq)


that handles the majority of the steps involved in the System Design Space method. It calculates the maximum number of phenotypes, constructs objects that represent qualitatively-distinct phenotypes, and provides utility functions for visualization of the system design space. By convention, the DesignSpace object that represents the biochemical system being analyzed is named ds—a short name for convenience because it is the starting point for so many analyses.

### Enumeration of the phenotypic repertoire

As mentioned in the previous sub-section, the DesignSpace object for a particular system is the starting point for most analyses. Among these analyses, perhaps the most important, is automatic enumeration of the phenotypic repertoire for a system. This is achieved by instructing the ds object to identify all its valid cases,


ph = ds.valid_cases()


where the output, stored in the “ph” variable, contains a list of case numbers for all the cases that have a feasible region somewhere in parameter space. These represent the qualitatively-distinct phenotypes of the system and together they define its phenotypic repertoire.

It should be noted that the ds object enumerates the phenotypic repertoire in parallel by creating a pool of cases that need to be tested, spawning *n* threads (where *n* is the number of processors available) that each request a case from the pool, and analyzing each case for validity. The results from each thread are returned to the ds object so it can assemble, sort and return the results. It should be noted that this is typically one of the most costly operations in an analysis and the results are stored by the ds object to eliminate excessive work following consecutive calls.

This can be applied to the example system and the number of valid phenotypes counted,


len(ph)


to show that it has a total of 10 qualitatively-distinct phenotypes that are valid somewhere in design space, as shown by the cases in Table 1.

**Table 1:** **Enumeration of the phenotypic repertoire for the simple system shown in Figure 1**.

**Case number**	**Case signature**	**∂ log X_1_/∂ log X_2_**	**∂ log X_1_/∂ log X_3_**	**Stability**
1	[1111]	0.0	0.0	S
4	[1211]	−1.0	0.0	S
7	[2111]	0.0	0.0	U
8	[2121]	0.0	0.0	S
9	[2131]	0.0	1.0	U
10	[2211]	1.0	0.0	U
11	[2221]	−1.0	0.0	S
12	[2231]	1.0	1.0	U
15	[3131]	0.0	0.0	S
18	[3231]	−1.0	0.0	S

### Phenotypic characteristics of qualitatively-distinct phenotypes

The qualitatively-distinct phenotypes of the system can be analyzed for a variety of system properties, or phenotypic characteristics, such as those previously discussed in Section Mathematical Definition of Qualitatively-Distinct Phenotypes. Many phenotypic characteristics are automatically determined by the toolbox and these are typically determined by analyzing instances of the Case class that represent different cases of the design space. Instances of the Case class are obtained by calls to the ds object using a case identifier. For example, we can create a Case object representing Case 1 by calling the ds object with the case number 1 (or ‘1’),


case1 = ds(1)


or with the case signature [1111],


case1 = ds(‘1111’, by_signature=True)


The phenotypic characteristics of a qualitatively-distinct phenotype typically fall within one of two categories: characteristics of the phenotype in the context of system design space (e.g., the boundaries of validity and global tolerance of the system to large qualitative changes; Coelho et al., 2009) or characteristics of the phenotype as they pertain to sub-system behavior (e.g., stability of the steady state and local robustness (insensitivity) of the system to small quantitative changes). In particular, our methods provide a novel means of characterizing global robustness, which we term “global tolerance” to clearly distinguish it from local robustness. Global tolerance is defined as the largest change in parameter values before there is a qualitative change in the phenotype (Coelho et al., 2009). This is determined automatically for each parameter and phenotype. Local robustness also is determined automatically by means of conventional parameter (in)sensitivities (local relative derivatives) for each parameter and phenotype. Some examples showing how this information is utilized in a stochastic context can be found in Fasani and Savageau (2013, 2015).

In general, characteristics in the context of system design space are determined from the Case object, and characteristics in terms of sub-system behavior can be acquired from instances of the SSystem class that represent the dominant S-System of a particular case. The SSystem object representing the dominant S-System for a case is a property of the Case object. For example, the SSystem instance of Case 1 can be retrieved by


ssys = case1.ssystem


and the properties of the dominant S-System can be readily determined. For example, we can view the equations of the dominant S-System,


ssys.equations


which returns


[X1.=X100^-1*a1-X1*b1, X100=1]


a list of Expression objects represented using strings. Similarly, the steady-state solution for the SSystem object can also be viewed using the


ssys.solution


command that returns


[X1=a1*b1^-1, X100=1]


The SSystem class can be used to show (1) the sub-system equations, (2) analytical steady-state solution—in Cartesian and logarithmic coordinates, (3) numerical values for the steady-state solution at a given point, (4) the steady-state fluxes at a given point, (5) local factors like logarithmic gains for signal amplification and parameter sensitivities for local robustness, and (6) information concerning the local stability of the system.

In the last three columns of Table 1 we show the logarithmic gains for *X*_1_ with respect to *X*_2_ and *X*_3_ and the stability of a representative fixed point for a particular case. These two types of characteristics are acquired for the sub-system from the SSystem object. We begin by showing how an instance of the SSystem class can be analyzed for its phenotypic characteristics.

#### Automated analysis of log-gain factors and parameter sensitivities

The logarithmic gains and parameter sensitivities are purely a function of the kinetic orders of an S-System (Savageau, 2009). In the context of System Design space, the kinetic orders are architectural features of the system and thus for any particular system design are assumed to be constant (Lomnitz and Savageau, 2015); therefore, the log-gain factors and parameter sensitivities are constant for a particular dominant S-System. Furthermore, because independent variables and parameters of the system are treated equally, the parameter sensitivities are obtained in the same way as log-gain factors.

The logarithmic gain of *X*_1_ relative to *X*_3_ for Case 1 of the example is determined by


ssys.log_gain(‘X1’, ‘X3’)


which, as shown in Table 1, is equal to 0—indicating that *X*_1_ is uncoupled from *X*_3_ and thus a change in *X*_3_ does not elicit a change in *X*_1_ as long as Case 1 is the qualitatively-distinct phenotype of interest.

#### Automated analysis of local stability

As discussed previously, the SSystem class automates the determination of local stability for the S-Systems. However, for conventional eigenvalue analysis, the first step involves converting the dominant S-Systems into a purely dynamical system by removing any algebraic constraints. This is necessary because most dominant S-Systems include algebraic constraints originating from the recasting process. The algebraic constraints can typically be removed by virtue of the fact that S-System equations have tractable steady-state solutions; hence, the auxiliary variables can be solved in terms of the dynamic variables and system parameters, and their solution can then be used to eliminate the algebraic constraints. Here, we show how a solution for the auxiliary variables can be determined using the algebraic constraints to create a new representation of the dominant S-System that is purely dynamical.

##### Removing algebraic constraints from differential-algebraic S-Systems

We begin with the equations for a dominant S-System composed of ODEs and algebraic constraints, as shown in Equations (10) and (11). The algebraic constraints, shown in Equation (11), are equivalent to *n*_*c*_ equations of an S-System at steady state, where *n*_*c*_ is the number of auxiliary variables. The steady-state solution for the S-System equations can be readily obtained by transforming the system into logarithmic coordinates (Savageau, 2009) that are represented in matrix notation by the following equation

(16) $$\begin{array}{r} Gy+a=Hy+b \end{array}$$

where *G* and *H* are *n*_*c*_ × (*n* + *m*) matrices of kinetic orders for the positive and negative terms of the algebraic equations, respectively—such that *G*_*ij*_ = *g*_*ij*_*p*_*i*___ and *H*_*ij*_ = *h*_*ij*_*q*_*i*___; *y* is an (*n* + *m*)-column vector—such that *y*_*i*_ = log*X*_*i*_; *a* and *b* are *n*_*c*_-column vectors of the logarithm of the rate constants for the positive and negative terms of the algebraic equations—such that *a*_*i*_ = logα_*i*_*p*_*i*___ and *b*_*i*_ = logβ_*i*_*q*_*i*___.

Next, we partition the *G* and *H* matrices into sub-matrices corresponding to dynamic, auxiliary and independent variables, represented by the *t, c* and *I* subscripts. Likewise, we partition the *y* vector into vectors corresponding to dynamic, auxiliary and independent variables,

(17) $$\begin{array}{r} y=\left[\begin{array}{c} y_{t}\\ y_{c}\\ y_{I} \end{array}\right] \end{array}$$

(18) $$\begin{array}{r} G=\left[\begin{array}{cc} G_{t}G_{c} & G_{I} \end{array}\right] \end{array}$$

(19) $$\begin{array}{r} H=\left[\begin{array}{cc} H_{t}H_{c} & H_{I} \end{array}\right] \end{array}$$

which yields the following system of equations in matrix notation,

(20) $$\begin{array}{r} G_{t}y_{t}+G_{c}y_{c}+G_{I}y_{I}+a=H_{t}y_{t}+H_{c}y_{c}+H_{I}y_{I}+b \end{array}$$

We rearrange the terms so that the auxiliary variables are on the left-hand side of the equation and all other variables are on the right-hand side,

(21) $$\begin{array}{r} A_{c}y_{c}=-A_{t}y_{t}-H_{I}y_{I}+B \end{array}$$

where *B* = *b* − *a*, and *A*_*i*_ = *G*_*i*_ − *H*_*i*_ for *i* = {*t, c, I*}.

We find the inverse of *A*_*c*_, defined as *M*_*c*_, and multiply both sides of the equation, which yields the following equation for the auxiliary variables,

(22) $$\begin{array}{r} y_{c}=-Ry_{t}-Sy_{I}+U \end{array}$$

where *R* = *M*_*c*_ *A*_*t*_ is an *n*_*c*_ × *n*_*t*_ matrix; *S* = *M*_*c*_ *A*_*I*_ is an *n*_*c*_ × *m* matrix; and *U* = *M*_*c*_*B* is an *n*_*c*_-column vector. The solution for the *i*-th auxiliary variables, in Cartesian coordinates, is therefore

(23) $$\begin{array}{r} X_{i}=u_{i}\overset{n_{t}}{\underset{j=1}{\prod }}X_{j}^{r_{ij}}\overset{n+m}{\underset{j=n}{\prod }}X_{j}^{s_{ij}} \end{array}$$

where *u*_*i*_ is the entry in the *i*-th row of the *U* vector; *r*_*ij*_ is the entry in the *i*-th row and (*j*–*n*_*t*_)-th column of the *R* matrix; and *s*_*ij*_ is the entry in the *i*-th row and (*j*–*n*)-th column of the *S* matrix.

Substituting the solution for the auxiliary variables into the dynamic equations of the dominant S-System yields the following system of *n*_*t*_ ODEs,

(24) $$\begin{array}{r} \begin{array}{cc} \frac{dX_{i}}{dt}= & \alpha_{ip_{i}}\overset{n_{c}}{\underset{j=1}{\prod }}u_{j}^{g_{ijp_{i}}}\overset{n_{t}}{\underset{j=1}{\prod }}X_{j}^{g_{ijp_{i}}-\overset{n}{\underset{k=n_{t}}{\sum }}g_{ikp_{i}}r_{kj}}\overset{n+m}{\underset{j=n}{\prod }}X_{j}^{g_{ijp_{i}}-\overset{n}{\underset{k=n_{t}}{\sum }}g_{ikp_{i}}s_{kj}}\\ -\beta_{iq_{i}}\overset{n_{c}}{\underset{j=1}{\prod }}u_{j}^{h_{ijq_{i}}}\overset{n_{t}}{\underset{j=1}{\prod }}X_{j}^{h_{ijq_{i}}-\overset{n}{\underset{k=n_{t}}{\sum }}h_{ikq_{i}}r_{kj}}\overset{n+m}{\underset{j=n}{\prod }}X_{j}^{h_{ijq_{i}}-\overset{n}{\underset{k=n_{t}}{\sum }}h_{ikq_{i}}s_{kj}} \end{array} \end{array}$$

which no longer has algebraic constraints and thus can be analyzed using conventional S-System analysis for local stability (Savageau, 2009).

To remove algebraic constraints for an instance of the SSystem class, we use the following command,


alt_ssystem = ssys.remove_algebraic_
              constraints()


which creates a new instance of the SSystem class that is equivalent mathematically, but does not have the auxiliary variables and associated algebraic constraints. The equation of the SSystem object representing the dominant S-System for Case 1, without the algebraic constraints, is


[X1.=a1-X1*b1]


##### Analyzing purely dynamical S-Systems for local stability

The SSystem object, without algebraic constraints, is then analyzed for its local stability using one of two methods: standard eigenvalue analysis or by applying the Routh criteria for stability (Routh, 1877; Yang, 2002). In either case, the stability of a dynamical system depends on a particular set of values for the parameters.

Starting with a reference parameter set, stored in the pvals variable of the VariablePool class, that represents a point in design space for the S-System, we determine the eigenvalues for the system,


alt_ssystem.eigenvalues(pvals)


or we can quickly get the number of eigenvalues with positive real part,


alt_ssystem.positive_roots(pvals)


The stability of Case 1, at this representative point, is stable, as shown in Table 1, given that it has 0 eigenvalues with positive real part.

#### Automated analysis of global tolerance

The Global Tolerance for a parameter and phenotype can be determined automatically from the Case object. From a geometric perspective, the global tolerance from a point is defined as the distance to the nearest boundaries of the enclosing phenotypic region in logarithmic coordinates (Fasani and Savageau, 2010). This can be calculated by performing a series of 1-D linear programming problems where all the parameters are fixed except for the parameter of interest.

The complete set of global tolerances for the example Case 1 at the starting reference parameter set is determined by


tolerances = Case1.measure_tolerances(pvals)


which returns a dictionary of key-value pairs, where the keys are the names of the parameters and the values are tuples with fold-decrease and fold-increase values representing the global tolerances in arithmetic coordinates, such that


tolerances[‘X3’]


yields the tuple (1e-20, 10.0). The first value indicates that a fold-decrease of 20 orders of magnitude are necessary to elicit a qualitative change in system behavior, whereas a 10 fold-increase results in a qualitative change in system behavior. Note that the value of 1e-20 is in fact bounded by the program, and typically corresponds to an infinitely large global tolerance—hence a qualitative change in system behavior cannot be achieved by only decreasing the value of *X*_3_. Other large but fixed values may be determined by physical constraints such as the solubility limits for a metabolite or the diffusion limit for a particular rate constant. The set of global tolerances for Case 1, given a representative interior point, are shown in Table 2.

**Table 2:** **Global tolerances for Case 1 of the system in Figure 1 measured as the fold-difference for a qualitative change in phenotype**.

**Parameter**	**Global tolerance**	
	**Fold-decrease**	**Fold-increase**
α_1_	1 × 10^−20^	3.162
β_1_	0.316	1 × 10^20^
ρ_1_	0.1	10.0
*K*_1_	0.316	1 × 10^20^
*K*_3_	0.1	1 × 10^20^
*k*	1 × 10^−20^	10.0
*X*_2_	1 × 10^−20^	10.0
*X*_3_	1 × 10^−20^	10.0

For this property, as for local stability in the previous section, starting from a representative point begs the question: How do we find this representative point?

### Predicting phenotype-specific parameter sets

One of the challenges when analyzing non-linear systems is finding parameter values that realize a particular behavior or, in the context of the System Design Space method, a particular qualitatively-distinct phenotype of the system. For example, this mathematical model has a total of 12 independent variables/parameters that together define a 12-dimensional space. The naive approach might be to sample this space to try and find a combination that yields a particular phenotype of interest. However, even if we were to sample five values for each of the 12 parameters, the number of combinations we would have to test would be enormous—5^12^ = 244,140,625; thus, this approach to search for values that realize a phenotype of the system is not feasible for most biological systems that have many more independent variables/parameters.

We have recently developed methods within the framework of the System Design Space approach that automatically predict representative values for any phenotype of the system (Lomnitz and Savageau, 2015). This is automated by the Design Space Toolbox V2 using linear programming techniques that can quickly and efficiently find the solution for the optimization of a linear function within a feasible region delimited by linear bounds (Vanderbei, 1996). Our software tools predict a set of values for the parameters of Case 1 using a simple instruction,


pvals = case1.valid_parameter_set()


that results in a parameter set near a vertex of the feasible region. Alternatively, parameter sets within the interior of the feasible region of a phenotype can be obtained by a variety of methods (e.g., see Lomnitz and Savageau, 2015) and is done using the following command:


pvals = case1.valid_interior_parameter_
         set()


The results for the local stability of the qualitatively distinct phenotypes shown in the last column of Table 1 were determined by predicting a set of parameter values in the interior and calculating the number of eigenvalues with positive real part. The particular parameter set predicted for Case 1 is: *K*_1_ = 1.00; *K*_3_ = 10.00; *X*_2_ = 1.00; *X*_3_ = 1.00; α_1_ = 1.00; β_1_ = 10.00; ρ_1_ = 10.00.

The possible sets of values that our tools can predict are effectively limitless. To focus the choices, we can (1) impose power law constraints on the dependent and independent variables of the system, (2) optimize a power law objective function, and (3) impose bounds on the permissible values for each of the parameters and independent variables. Each of these options is a simple command, e.g.,


case1 = ds(1, constraints=[‘X1 > 100’])
pvals = case1.valid_parameter_set
        (p_bounds={‘X3’:[1e-3, 1e3]},
optimize=‘X1^2*X2^2*X3^-2’
  
)


### Predicting ensemble-specific parameter sets

We have previously developed methods that enable the prediction of parameter values for the simultaneous realization of an ensemble of model phenotypes (Lomnitz and Savageau, 2015). The types of ensembles for which these tools can predict a corresponding set of parameter values fall within three categories: (1) intersections of phenotypes at a single point in design space; (2) co-localization of phenotypes within a slice of design space; and (3) arrangement of phenotypes phased within a slice of design space to exhibit a particular progression of behaviors.

#### Predicting parameter sets for case intersections

The validity of the intersection of multiple cases in design space can be readily determined using linear programming methods (Fasani and Savageau, 2010). This is achieved by combining the *N*_*i*_ boundary conditions of *n* different qualitatively-distinct phenotypes (Fasani and Savageau, 2010). This is particularly useful to determine if a system can exhibit multi-stability such as bistable regimes for hysteretic switches. The Design Space Toolbox V2 extends the analysis of intersecting cases beyond determining their validity. It enables prediction of values for the parameters that yield such an intersection. We begin by defining an object of the CaseIntersection class, that inherits many of its properties from the Case class.

Using the example DesignSpace object defined in Construction of the System Design Space, with the phenotypic repertoire shown in Table 1, we can determine if the intersection of different ensembles of cases are mathematically possible and, if so, predict values for the parameters that lead to their realization. To illustrate the CaseIntersection class, we apply it to identify intersections of three phenotypes consistent with bistable regimes. We choose the first two stable cases, Cases 1 and 4, and the first unstable case, Case 7, as shown in the last column of Table 1,


case1, case4, case7 = ds([1, 4, 7])
  
in = dspace.CaseIntersection([case1, case4,
      case7])


With the CaseIntersection object, we can determine validity as if it were a Case object by using the following command,


in.is_valid()


which yields False. This indicates that the intersection of Cases 1, 4, and 7 does not exist - regardless of values for the parameters. We can select an alternative intersection of three cases by selecting the next possible stable case, Case 8, instead of Case 4,


case8 = ds(8)
  
alt = dspace.CaseIntersection([case1,
      case7, case8])


and we determine its validity,


alt.is_valid()


which yields True. This indicates that the intersection of Cases 1, 7, and 8 exists and we can now proceed to predict a set of values for the parameters that results in the realization of this intersection. As with the Case object, we can predict a set of values using the valid_parameter_set method or valid_interior_parameter_set method,


pvals = alt.valid_interior_parameter_set()


which yields the following set of values for the parameters: *K*_1_ = 1.00; *K*_3_ = 10.00; *X*_2_ = 1.00; *X*_3_ = 1.00; α_1_ = 0.32; β_1_ = 10.00; ρ_1_ = 100.00.

#### Predicting parameter sets for case Co-localizations

An extension of the Case Intersection concept is Case Co-localization. This concept involves identifying an ensemble of *n* phenotypes that are simultaneously realized, hence they are valid, within a *slice* of design space for which a given number of parameters or independent variables are allowed to change. These variables, known as slice variables, define an *s* dimensional slice through design space, where *s* is the number of slice variables (Lomnitz and Savageau, 2015). The qualitatively distinct phenotypes, Cases as we have defined them, are the phenotypes associated with parameter values located within a particular polytope in system design space. Such polytopes may abut one another, or they might be completely separated; the situation is difficult to visualize in a high-dimensional space. The objective of the case co-localization function is to find a set of parameter values, if it exists, that yields a “slice” through the high-dimensional space that allows simultaneous visualization of selected polytopes. An intuitive example would be to determine if two phenotypes, e.g., a wild type and diseased phenotype, are simultaneously realized and the transition visualized within a 2D slice, where one axis represents a genotypically determined parameter and the other an environmentally determined variable.

We have previously shown that the validity of case co-localizations can be determined without sampling parameter space, it can be used for an arbitrary number of phenotypes and can be done in an arbitrary number of dimensions (Lomnitz and Savageau, 2015). We begin by duplicating and renaming the slice variables for each case in the ensemble and combining the boundaries for each case with the newly defined variables (Lomnitz and Savageau, 2015). The result is an (*m* + *n* (*s* − 1))-dimensional convex polytope in logarithmic space, where *s* is the number of slice variables that can be analyzed in the same way as the feasible regions for Cases and Case Intersections.

The CaseColocalization class inherits properties from the CaseIntersection class and can be analyzed in the same way as a CaseIntersection object. As an example, we define an ensemble of phenotypes, composed of cases 8, 12, 15, and 18 and apply methods similar to those described in the previous sub-section. This ensemble for co-localization, with *X*_2_ as the slice variable, is created as follows:


c8,c12,c15,c18 = ds([8, 12, 15, 18])
  
co = dspace.CaseColocalization([c8, c12,
     c15, c18], [‘X2’])


With the CaseColocalization object, we can determine validity of the ensemble by using the following command:


co.is_valid()


In this example it yields True. This indicates that there is a simultaneous realization of these behaviors within a slice of parameter space, and that there are sets of values for the parameters capable of realizing this ensemble. Moreover, the sets can be determined automatically by using the valid_parameter_set method or valid_interior_parameter_set method,


co.valid_interior_parameter_set()


which yields the following sets of values for the parameters: *K*_1_ = 1.00; *K*_3_ = 0.10; *X*_3_ = 1.00; α_1_ = 0.10; β_1_ = 10.00; ρ_1_ = 10000.00; *X*_2, 8_ = 1.00; *X*_2, 12_ = 100.00; *X*_2, 15_ = 1.00; *X*_2, 18_ = 100.00—where *X*_2, *i*_ represents the value for the *X*_2_ variable within the feasible region for Case *i*.

#### Predicting parameter sets for specific arrangements of cases

The method of case co-localization determines if an ensemble of cases can be simultaneously realized within some *s*-dimensional slice of parameter space, automatically and independent of sampling this infinitely large space. However, it does not yield any information about how the cases in the ensemble are located in the *s*-dimensional slice relative to each other or other important landmarks in the system design space. However, because these co-localizations are extensions of the methods that analyze cases in design space, we can apply the same methods. In particular, recall that the validity of cases in design space can be determined within particular constraints, as shown briefly at the end of Predicting Phenotype-Specific Parameter Sets. These same methods can be applied to objects of the CaseColocalization class to achieve a particular progression of behaviors (Lomnitz and Savageau, 2015), by imposing a set of power law constraints among all variables including replicated slice variables.

To illustrate this, we will create an ensemble of cases 8, 12, 15, and 18 arranged in ascending numerical order, such that *X*_2, 8_ < *X*_2, 12_ < *X*_2, 15_ < *X*_2, 18_, are located from left to right in the design space of the system. We can determine whether this arrangement is possible somewhere in parameter space and predict values for the parameters that yield this particular arrangement.

An arrangement is created in the Design Space Toolbox V2 by introducing a new co-localization and adding constraints between the replicated slice variables representing *X*_2_ when defining the co-localization. The replicated slice variables representing *X*_2_ are defined with special notation using the following format: $<slice variable>_<index in colocalization>. In this example, the slice variable is *X*_2_ and the indices in the co-localization for cases 8, 12, 15, and 18 are 0, 1, 2, and 3, respectively. Thus, the arrangement is constructed by


c8,c12,c15,c18 = ds([8, 12, 15, 18])
  
arr = dspace.CaseColocalization
      ([c8, c12, c15, c18],
  
[‘X2’],
  
constraints =[‘$X2_0  <  $X2_1’,
  
            ‘$X2_1  <  $X2_2',
  
            ‘$X2_2  <  $X2_3’,
  
)


The arrangement is simply a Case Co-localization plus additional constraints; thus, we determine validity and predict values for the parameters in the same way as we did for co-localization,


arr.is\_valid()


which yields False. The result is that the specific arrangement that we specified is not possible, regardless of values for the parameters. Using the same cases, we can try different relative arrangements and additional constraints as long as both sides of the inequality defining the constraints are power laws. As an example, consider another arrangement involving Cases 8, 12, 15, and 18 with a different order by flipping the sign for one of the inequalities,


c8,c12,c15,c18 = ds([8, 12, 15, 18])
  
arr = dspace.CaseColocalization
      ([c8, c12, c15, c18],
  
[‘X2’],
  
constraints = [‘$X2_0  <  $X2_1’,
  
            ‘$X2_1 > $X2_2’,
  
            ‘$X2_2  <  $X2_3’,
  
            ‘$X2_1 > $X2_3’]
  
)


The validity of this co-localization yields True and thus, we can predict a set of values that realizes this arrangement: *K*_1_ = 1.00; *K*_3_ = 0.10; *X*_3_ = 1.00; α_1_ = 0.01; β_1_ = 1.00; ρ_1_ = 10000.00; *X*_2, 8_ = 0.10; *X*_2, 12_ = 10.00; *X*_2, 15_ = 0.10; *X*_2, 18_ = 10.00.

### Visualizing the design space of biochemical systems

One of the powerful features of the System Design Space method is that it partitions parameter space into regions and this structure reveals breakpoints in the characteristics of the system. This structured space, the design space of a system, can readily be visualized to gain insight into the landscape of possible phenotypes that a system can exhibit. These software tools enable visualization through the matplotlib package, the NumPy package and the SciPy package (Oliphant, 2007; Millman and Aivazis, 2011; van der Walt et al., 2011). We illustrate the Design Space Toolbox V2 visualization tools by applying them to the example system using the parameter set from the intersection of Cases 1, 7, and 8. In addition, we show the visualization of the stability showing the number of eigenvalues with positive real part.

The first step is to import the matplotlib plotting package into the python environment,


from matplotlib.pyplot import *


and import the plotting extension for the dspace classes,


import dspace.plotutils


The typical way of visualizing a design space is by showing the qualitatively-distinct phenotypes in a 2D plot, where the *x-* and *y*-axes represent slice variables and the z-axis represents different cases identified by different colored regions.

**Figure 3:**
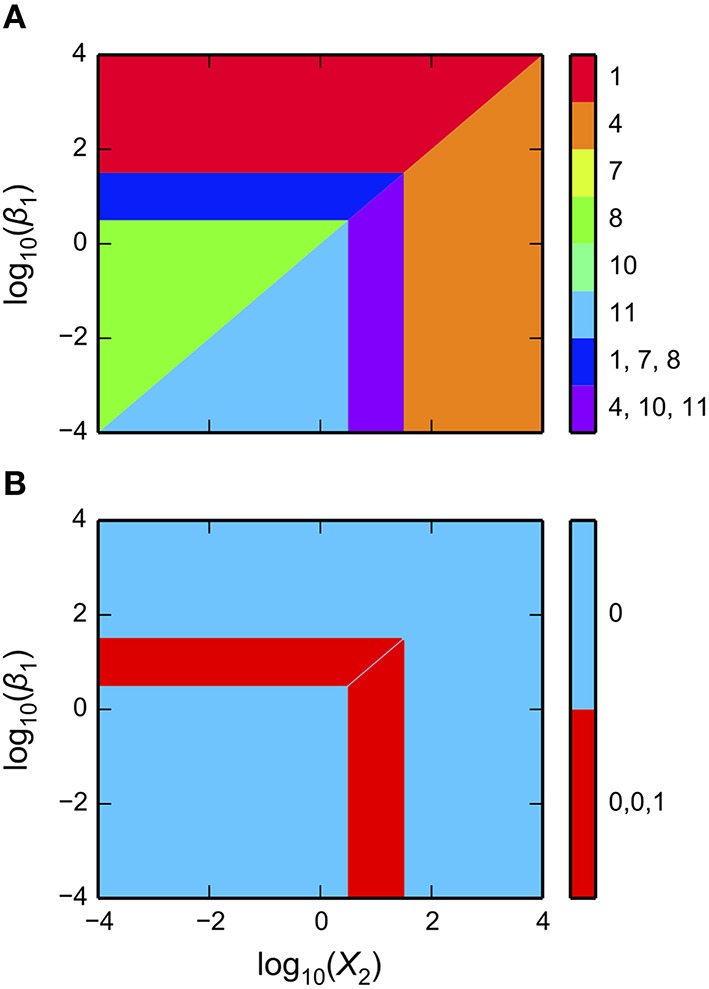
**Visualization of the system design space and a phenotypic trait for the simple synthetic gene circuit in Figure 1**. **(A,B)** The *x*-axis represents the concentration of the complimentary protein, *X*_2_. The *y*-axis represents the rate constant for *X*_1_ loss from either dilution or active degradation. **(A)** System design space showing the qualitatively-distinct phenotypes by color on the *z*-axis. Regions of overlap, represented by regions with multiple qualitatively-distinct phenotypes as shown in the colorbar, correspond to regions with multiple fixed points. **(B)** Stability plot showing the number of eigenvalues with positive real part on the *z*-axis. Blue corresponds to monostability; Red corresponds to bistability. Note that the regions of bistability in **(B)** correspond to the regions of overlap in **(A)**.

Using the example DesignSpace object from the previous sub-sections, and the parameters predicted for the intersection of Cases 1, 7, and 8, these tools create the plot of the 2-D slice by the command


ds.draw_2D_slice(gca(), #:1
  
                 pvals, #:2
  
                 ‘X2’,  #:3
  
                 ‘b1’,  #:4
  
           [1e-3, 1e3], #:5
  
           [1e-3, 1e3], #:6
  
intersections = [1, 3] #:7
  
)


as shown in Figure 3A. The first argument is a matplotlib axis object for a plot canvas; the second argument is an instance of the VariablePool class with the values for the parameters; the third is the name of the *x*-axis; the fourth argument is the name of the *y*-axis; the fifth argument is the range of the *x*-axis in Cartesian coordinates; the sixth argument is the range of the *y*-axis in Cartesian coordinates; the seventh argument indicates the number of intersections of cases to be drawn, where [1, 3] indicates it will display regions associated with individual phenotypes and with three phenotypes consistent with bi-stability (i.e., 2 stable and 1 unstable).

The stability of the fixed points also can be visualized. This is achieved by using a different command, but with mostly the same set of arguments,


ds.draw_2D_positive_roots(gca(), #:1
  
                          pvals, #:2
  
                           ‘X2’, #:3
  
                           ‘b1’, #:4
  
                    [1e-3, 1e3], #:5
  
                    [1e-3, 1e3], #:6
  
)


as shown in Figure 3B.

The toolbox provides additional tools to visualize dominant eigenvalues, steady-state concentrations, steady-state fluxes, and mathematical functions evaluated at steady state. It also provides tools to visualize 1-D slices and 1-D response curves including stability information for bifurcation plots.

## Example applied to a synthetic memory module

In this Section, we illustrate the general capabilities of the Design Space Toolbox V2 by applying it to a two-gene synthetic circuit involving two transcriptional activators. This example serves the dual purpose of highlighting the novel, phenotype-centric, modeling strategy we have recently developed that inverts many of the typical steps in the conventional, parameter-centric, modeling approach (Lomnitz and Savageau, 2015).

The novel modeling approach begins by enumerating the phenotypic repertoire for a global perspective of system behavior; then predicting phenotype-specific or ensemble-specific parameter sets that realize phenotypic characteristics of interest; and finally focusing computational effort on localized regions of the parameter space for detailed analysis of the full system. The Design Space Toolbox V2 enables this novel modeling approach by automating the most difficult steps in the process.

The synthetic gene circuit, proposed and analyzed here, is intended to serve as a genetic hysteretic switch that can exhibit multistability. We show that this circuit can “count” between three distinct states in a positive direction that increases the counter and in a negative direction that decreases the counter. We show that by coupling the circuit with a target gene, a reporter, it can transition between three distinct intensity levels in a step-wise manner.

In the following sub-sections we (1) describe the design of the synthetic gene circuit; (2) formulate a mathematical model that captures the mechanistic details of the interactions; (3) analyze the system using our phenotype-centric modeling strategy; and (4) show examples of instances of the system at predicted points in the system's design space that exhibit a variety of behaviors.

### Synthetic gene circuit design

Synthetic gene circuits have been constructed to serve a variety of purposes (Lu et al., 2009). One prominent use for synthetic biology is to forward engineer biological systems to gain insight into fundamental design principles (Mukherji and van Oudenaarden, 2009). Some examples that apply principles from engineering to biological systems include rationally designed synthetic oscillators (Elowitz and Leibler, 2000; Atkinson et al., 2003; Tigges et al., 2009) and bistable switches (Gardner et al., 2000; Atkinson et al., 2003).

We apply similar principles for the design of a system with the potential to exhibit multistability. This implies that there are instances of the system that have multiple stable fixed points, also known as steady-state *attractors*, with an associated set of initial conditions that define the *basin of attraction* within which the system gravitates toward a particular fixed point in state space.

**Figure 4:**
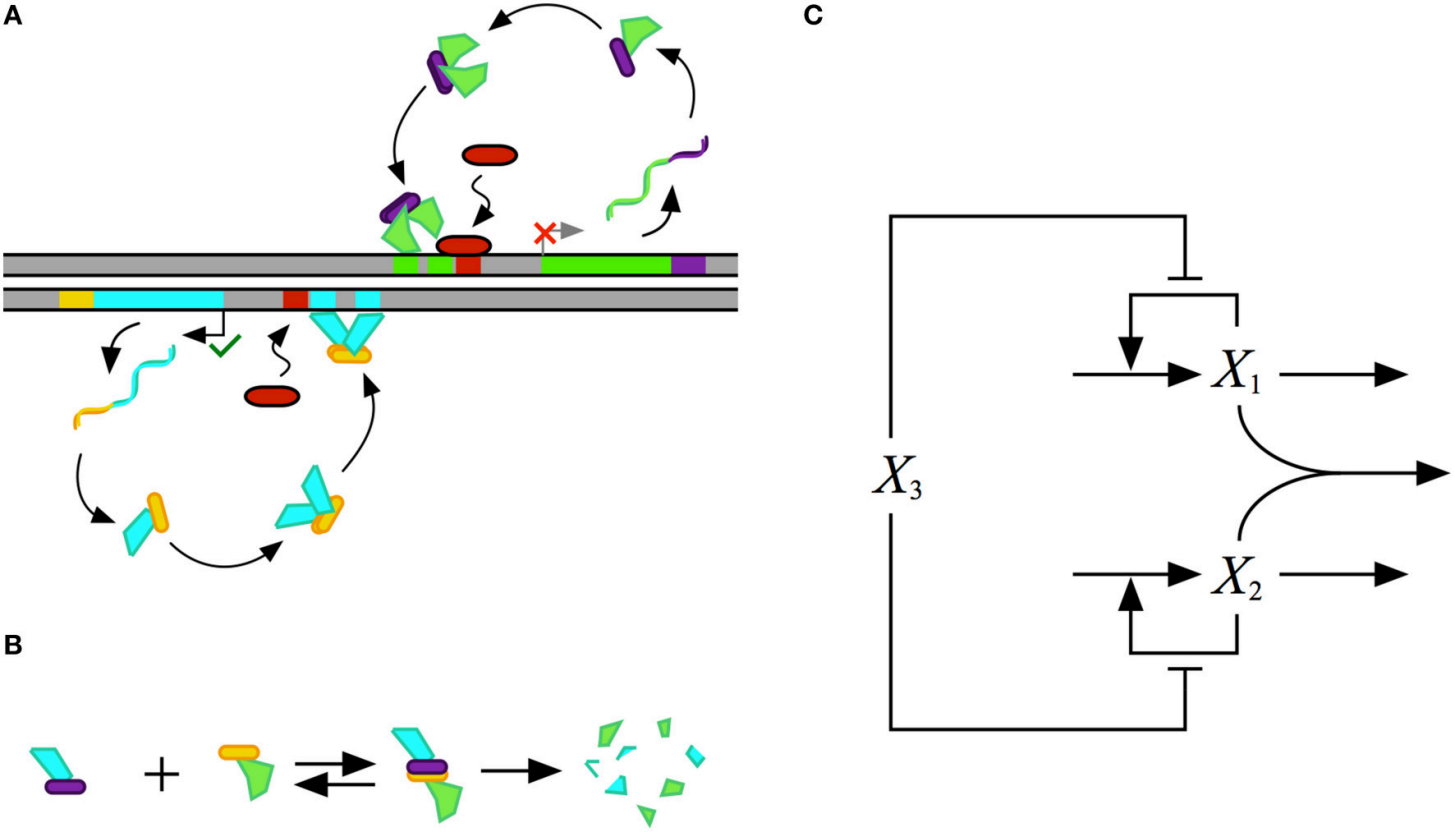
**Conceptual model for the design of a synthetic gene circuit with 2-, 3-, and 4-state memory**. **(A)** A cartoon of the proposed design for a gene circuit with two autogenously regulated activators, each similar to that in Figure 1. The first is represented in green with a purple dimerization domain and the second is represented in blue with a yellow dimerization domain. Homodimerization of each leads to the active form of the regulator. A repressor, represented by the red capsule, sterically hinders the binding of each activator. **(B)** Binding of monomers from each of the two activators through complementary dimerization domains leads to a heterodimer that is rapidly degraded by cellular proteases or other machinery. **(C)** Abstract representation of the synthetic construct. The two activators *X*_1_, green in the cartoon, and *X*_2_, blue in the cartoon, heterodimerise to create a complex that is degraded, each activates its own expression by binding to target DNA, and this binding is sterically hindered by the common repressor *X*_3_, red in the cartoon.

The design of the synthetic gene circuit, represented in Figure 4, is composed of two transcriptional activators, *X*_1_ and *X*_2_ that autogenously control expression of their own genes; the result is two seemingly independent positive feedback loops. The *X*_1_ and *X*_2_ regulators are translationally fused with a dimerization domain that causes *X*_1_ monomers to form heterodimers with *X*_2_ monomers. The *X*_1_–*X*_2_ dimers are inactive and targeted for degradation by cellular proteases, which results in a strong thermodynamic potential that makes heterodimer formation essentially irreversible. Transcription of the activator genes is repressed by a third regulator, *X*_3_, that binds to the upstream region of the gene for both *X*_1_ and *X*_2_, sterically hindering the auto-activation. The role of this repressor in the system is to tune the behavior of the system. A cartoon of the proposed construct is shown in Figure 4A, and an abstraction of the gene circuit with the key interactions is shown in Figure 4C.

### Mathematical model

We formulate a mathematical model composed of ODEs for the synthetic gene circuit design in Figure 4. Given that there is a fast turnover of mRNA relative to protein, we assume that synthesis of protein directly tracks mRNA expression. Thus, we model modulation of transcription as having a direct effect on the rate of protein synthesis. The mathematical model is described by the following system of non-linear equations,

(25) $$\begin{array}{r} \frac{dX_{1}}{dt}=\alpha_{1}\left[\frac{1+\rho_{1}{\left(\frac{X_{1}}{K_{1}}\right)}^{2}+\frac{X_{3}}{K_{3}}}{1+{\left(\frac{X_{1}}{K_{1}}\right)}^{2}+\frac{X_{3}}{K_{3}}}\right]-\beta_{1}X_{1}-kX_{1}X_{2} \end{array}$$

(26) $$\begin{array}{r} \frac{dX_{2}}{dt}=\alpha_{2}\left[\frac{1+\rho_{2}{\left(\frac{X_{2}}{K_{2}}\right)}^{2}+\frac{X_{3}}{K_{3}}}{1+{\left(\frac{X_{2}}{K_{2}}\right)}^{2}+\frac{X_{3}}{K_{3}}}\right]-\beta_{2}X_{2}-kX_{1}X_{2} \end{array}$$

where α_*i*_ represents the basal level of expression for the synthesis of the *i*-th regulator; β_*i*_ represents the rate constant for loss of the *i*-th regulator by dilution due to exponential growth; ρ_*i*_ represents the capacity for activation by the *i*-th regulator; *K*_*i*_ represents the concentration of the *i*-th regulator for half-maximal regulation; and *k* represents the rate constant for *X*_1_–*X*_2_ heterodimer formation.

#### Recasting equations into the generic GMA form

We recast the mathematical model into the generic GMA form using the 5-step approach outlined in Recasting Equations into a Generic Form, which yields the following system of differential-algebraic equations,

(27) $$\begin{array}{rc} \frac{dX_{1}}{dt}= & \alpha_{1}X_{100}^{-1}+\alpha_{1}\rho_{1}X_{1}^{2}K_{1}^{-2}X_{100}^{-1}+\alpha_{1}X_{3}K_{3}^{-1}X_{100}^{-1}\\ -\beta_{1}X_{1}-kX_{1}X_{2} \end{array}$$

(28) $$\begin{array}{rc} \frac{dX_{2}}{dt}= & \alpha_{2}X_{200}^{-1}+\alpha_{2}\rho_{2}X_{2}^{2}K_{2}^{-2}X_{200}^{-1}+\alpha_{2}X_{3}K_{3}^{-1}X_{200}^{-1}\\ -\beta_{2}X_{2}-kX_{1}X_{2} \end{array}$$

(29) $$\begin{array}{rc} 0= & 1+X_{1}^{2}K_{1}^{-2}+X_{3}K_{3}^{-1}-X_{100} \end{array}$$

(30) $$\begin{array}{rc} 0= & 1+X_{2}^{2}K_{2}^{-2}+X_{3}K_{3}^{-1}-X_{200} \end{array}$$

where *X*_100_ and *X*_200_ are the auxiliary variables defined for the denominators in Equations (25) and (26), respectively. These equations are then used as input to the Design Space Toolbox V2 for analysis as described in Section Construction of the System Design Space.

### Computer-aided novel modeling strategy

We analyze the system using the phenotype-centric modeling strategy (Lomnitz and Savageau, 2015) that involves (1) establishing criteria for what constitutes the model phenotypes of interest, (2) enumerating the repertoire of model phenotypes, (3) identifying model phenotypes that exhibit the characteristics of interest, and (4) predicting values for the parameters that realize the desired behavior. We have previously used this strategy to identify phenotypes that exhibit the potential for oscillation (e.g., see Lomnitz and Savageau, 2015) or specific couplings between inputs and outputs to achieve binary logic functions (Lomnitz and Savageau, in press). Here, the phenotype-centric modeling strategy is applied to identify a variety of phenotypes including bistability, tristability and quadrastability.

### Criteria for model phenotypes of interest

The first step in the phenotype-centric modeling strategy is to establish criteria for what constitutes a phenotype of interest based on a set of phenotypic characteristics. Typical characteristics include the coupling between input and output, stability of the fixed points, quantitative local robustness to small changes in system parameters, and qualitative global tolerance to large changes in system parameters.

The design for the synthetic gene circuit in Figure 4 is expected to have the potential to exhibit multistability; therefore, there should be multiple fixed points, some of which are stable and some unstable, at a single point in parameter space. In the context of a system's design space, multistability involves an overlap or intersection of multiple cases (Savageau and Fasani, 2009; Fasani and Savageau, 2010; Martínez-Antonio et al., 2012).

Although multistability involves a combination of cases exhibiting either unstable or stable fixed points, we are interested in those that are stable; thus, the first criterion for what constitutes a phenotype of interest is that it be locally stable. Furthermore, a desirable property is that the fixed points be locally insensitive to unintended signals; thus, a second and third criterion is that both *X*_1_ and *X*_2_ are uncoupled from the repressor, *X*_3_. In summary, we are looking for cases that are locally stable, have X_1_ uncoupled from X_3_ [L(X_1_, X_3_) = 0], and have *X*_2_ uncoupled from X_3_ [L(X_2_, X_3_) = 0].

### Enumerating the repertoire of phenotypes of interest

The mechanistic model for the synthetic gene circuit is analyzed here following the outline in Section Design Space Toolbox V2: we (1) refactor the system equation into the computer-readable format to construct a DesignSpace object, which we call ds (e.g., Section Construction of the System Design Space), (2) enumerate the valid phenotypes of the system using the ds.valid_cases() method (e.g., Section Enumeration of the Phenotypic Repertoire), and (3) determine the phenotypic characteristics of each valid phenotype to identify (a) the number of eigenvalues with positive real part at a representative point, (b) L(X_1_, X_3_), and (c) L(X_2_, X_3_) (e.g., Section Phenotypic Characteristics of Qualitatively-Distinct Phenotypes). The representative point to identify the number of eigenvalues with positive real part is predicted using the valid_interior_parameter_set() method of an instance of the Case class as described in Section Predicting Phenotype-Specific Parameter Sets. The number of phenotypes that satisfy our criteria are 21 of the 59 valid phenotypes, a portion of which is shown in Table 3.

**Table 3:** **Enumeration of the phenotypic repertoire for the system shown in Figure 2**.

**Case number**	**Case signature**	**L(X_1_, X_3_)**	**L(X_2_, X_3_)**	**Stability**
1	11111111	0.0	0.0	S
10	11121111	0.0	0.0	S
19	11211111	0.0	0.0	U
…	…	…	…	…
297	32213131	1.0	1.0	U
306	32223131	0.5	0.5	U
315	32313131	0.0	0.0	S

### Alternative realizations of the synthetic gene circuit

#### Maximizing the number of stable states

In Sections Predicting Phenotype-Specific Parameter Sets and Predicting Ensemble-Specific Parameter Sets we showed that our tools are able to predict values for the parameters that are specific to a phenotype or to an ensemble of phenotypes—either Case intersections at a single point in design space, Case co-localizations in a slice of design space, or Case specific arrangements in a slice of design space. Among these ensembles, Case intersections are particularly useful to identify the existence of multistability (Fasani and Savageau, 2010), and the ability of our tools to predict parameter values for their realization, as shown in Section Predicting Parameter Sets for Case Intersections, offers some interesting possibilities.

The first possibility we explore is the ability to identify the *maximum* number of stable phenotypes that can intersect in the system's design space, as this corresponds to the maximum number of steady-state attractors the system can exhibit. The general strategy on how to identify case intersections of *n* cases has been previously described (Fasani and Savageau, 2010). Here, we use this same approach but only apply it to the cases that are stable given that we are not interested in the cases that are unstable.

If the cases that satisfy the criteria are stored in the cases variable, our tools can list all the intersection of *k* = {2, 3, 4, …, *n*} cases. If for some value of *k* there are no intersections, the program stops and the value of *k*–1 is the maximum number of case intersections. The first step of finding all the intersections of *k* = {2, 3, 4, …, *n*} cases is achieved by


attractors = ds.intersecting_cases
            (range(2, 22),cases)


and the result is a list of all possible intersections involving combinations of 2 up to 21 cases. These are stored in the attractors variable and used to identify the largest number of intersecting cases,


max([len(i._cases) for i in attractors])


which yields a maximum of four cases with stable fixed points that can be simultaneously realized at a single point in design space. Therefore, this design for a genetic memory module can have up to four steady state attractors for quadrastablity.

#### Predicting parameter sets for realization of multi-stability

The gene circuit design has a maximum of four steady-state attractors in which *X*_1_ and *X*_2_ can be high or low at any given time. This result might not be surprising, given that the system has two positive feedback loops that appear to be independent from each other. However, these positive feedback loops are part of an integrated system and can interact to produce interesting behaviors. One could speculate that an increase in either *X*_1_ or *X*_2_ might lead to a decrease in *X*_2_ or *X*_1_, respectively, due to the formation of *X*_1_–*X*_2_ heterodimers. Here, we explore a series of alternative behaviors for bistable, tristable and quadrastable switches including a stable counter with three different levels.

**Figure 5:**
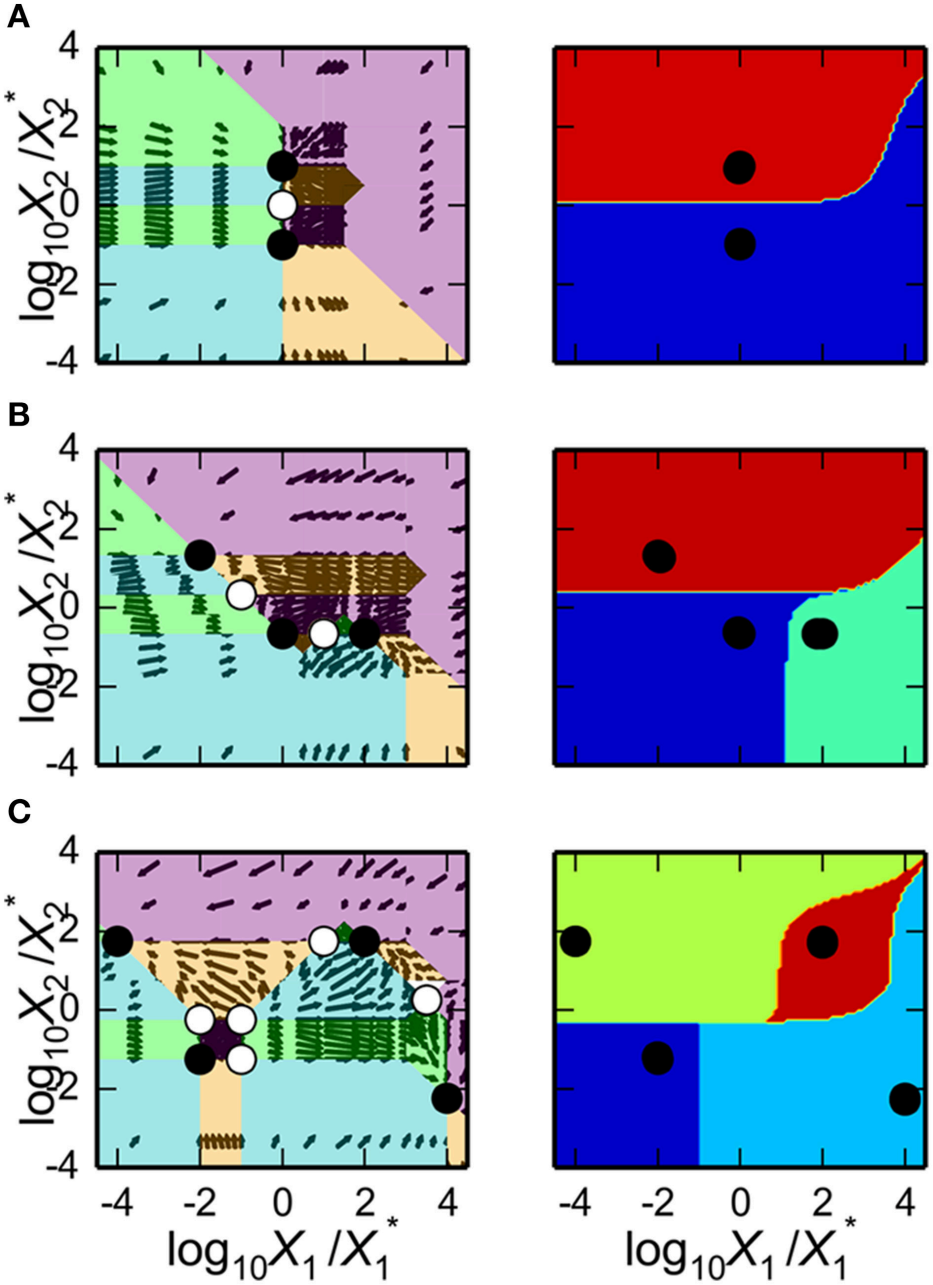
**Dynamic behavior for the bistable, tristable, and quadrastable instances of the synthetic gene circuit in Figure 4. (A–C)** The x-axis represents the logarithm of the concentration of the first activator, *X*_1_; the y-axis represents the logarithm of the concentration of the second activator, *X*_2_. The axes are normalized with respect to the mean of the values for *X*_1_ and *X*_2_ for each of the stable steady states in a given instance, respectively. The dynamic behaviors and basins of attraction for each of the stable states for instances of the system exhibiting **(A)** bistability, **(B)** tristability, and **(C)** quadrastability. The steady states of the system are represented by black circles (stable) and white circles (unstable). (Left panels) State-space deconstruction of the gene circuit by system design space showing qualitatively-distinct trajectories. Different colored regions represent areas where the dynamics of the system follow a particular trajectory: southwest (purple); southeast (green); northwest (orange); and northeast (blue). (Right Panels) Different colored regions represent values for the activators that are attracted to a unique steady-state (•). The boundaries between the basins of attraction are obtained by refinement using the original equations.

The System Design Space method we have described can be applied for a deconstruction of dynamic behaviors in state space. This deconstruction, which is still in the early phases of its development, partitions state space into regions that exhibit qualitatively-distinct trajectories that provide valuable information regarding the system's basins of attraction and response to transient perturbations. The dominance conditions define (*n* + *m*)-dimensional polytopes, where *n* is the number of dependent variables and *m* is the number of independent variables/parameters. For each equation in the Dominant S-System, we can identify regions where the positive term is greater than the negative term and thus a region with a qualitatively-defined trajectory. The particular arrangement of steady states and the trajectories around these steady states can be represented visually, as shown in the left panels of Figure 5, and can be compared with the basins of attraction for the original system of equations, as shown in the right panels of Figure 5. In each of these examples, our automated tools provide rich information for rapid identification of interesting properties for the system. The results can then be refined by applying conventional methods to the full system.

##### Predicting bistable genetic switches

The Design Space Toolbox V2 can be used to predict values for the parameters that result in instances of the system that are only bistable switches. We achieve this in two steps: we identify all the valid ensembles of two stable phenotypes satisfying our criteria, and then predict representative parameter values and identify those instances that have only two steady-state attractors—to eliminate ensembles that might be part of higher-order ensembles with more steady-state attractors.

The first step is most easily achieved using the same command as in Section Alternative Realizations of the Synthetic Gene Circuit, modified to return only Case Intersections involving two stable phenotypes,


en2 = ds.intersecting_cases([2],cases)


where en2 stores all the ensembles of two stable phenotypes at a single point.

The second step is achieved by iterating through each ensemble [for en in en2:]; predicting a representative point that realizes an ensemble [pvals=en.valid_ interior_parameter_set()]; identifying the cases valid at the representative point [all_cases = ds(ds.valid_cases(p_bounds=pvals))]; and counting the number of cases that are locally stable [sum([case.positive_roots() == 0 for case in all_cases])]. An example from among the six showing a bistable instance of the design, as predicted following these steps, is shown Figure 5A.

##### Predicting tristable genetic switches

We identify instances of the system that exhibit tristability using the same approach used to identify bistability—we identify the valid ensembles of three stable phenotypes and select those that have only three steady-state attractors. We change the first step by identifying the ensembles with Case Intersections of three stable phenotypes,


en3 = ds.intersecting_cases([3],cases)


and proceed with the same steps used for the bistable case. We find eight ensembles that exhibit tristability, an example of which is shown in Figure 5B.

##### Predicting quadrastable genetic switches

Because the maximum number of stable phenotypes that can intersect at a given point in design space is 4, the task of identifying instances of the system that exhibit quadrastability is simpler than the bistable and tristable examples. Here, all we need to do is identify ensembles of four stable phenotypes,


en4 = ds.intersecting_cases([4],cases)


which yields a total of 18 that can exhibit quadrastability. An example is shown in Figure 5C.

#### Predicting state-space arrangements of the steady-state attractors

As we discussed in Section Predicting Phenotype-Specific Parameter Sets, we can add constraints to the system and thus the number of parameter sets we can predict is effectively limitless. Here, we show how constraints can be impose to identify relative arrangements of the steady-state attractors that are permissible in state space. To achieve this, we define new independent variables that partition state space into four quadrants [i.e., (–,–), (−, +), (+, −), and (+, +)] and apply our tools to determine which combination of quadrants the stable-state attractors can occupy.

We define two variables, *X*_*r*_,_1_ and *X*_*r*_,_2_, that partition state space into the four quadrants with the boundaries *X*_1_ = *X*_*r*_,_1_ and *X*_2_ = *X*_*r*_,_2_, such that the (−, −) quadrant is given by *X*_1_ < *X*_*r*_,_1_ and *X*_2_ < *X*_*r*_,_2_. Then, we reconstruct a new instance of the DesignSpace class with the independent variables explicitly defined to include the *X*_*r*_,_1_ and *X*_*r*_,_2_ variables.

The new instance of the DesignSpace class can create new instances of the Case class with added constraints, as shown in Section Predicting Phenotype-Specific Parameter Sets. From Section Predicting Parameter Sets for Realization of Multi-Stability we identified all the ensembles of four stable phenotypes that result in a quadrastable instance of the system. We then test the validity of each of these ensembles with constraints imposed on its constitutive cases. For example, if we have four cases with case identifiers represented by the variables case0, case1, case2, and case3 that comprise an ensemble for a quadrastable system, we impose constraints on these cases to ensure that each is in a separate quadrant as follows,


C0 = ds(case0, constraints = [‘X1  <  Xr1’,
     ‘X2  <  Xr2’])
  
C1 = ds(case1, constraints = [‘X1  <  Xr1’,
     ‘X2 > Xr2’])
  
C2 = ds(case2, constraints = [‘X1 > Xr1’,
     ‘X2  <  Xr2’])
  
C3 = ds(case3, constraints = [‘X1 > Xr1’,
     ‘X2 > Xr2’])
  
ensemble = space.CaseIntersection([C0,
           C1, C2, C3])


and validity of the ensemble can be tested as shown in Section Predicting Ensemble-Specific Parameter Sets. We apply this to test each of the 35 combinations of criteria like that in the example above. We find that 24 of the 35 can satisfy their relevant criteria and that the remaining 11 are unable to satisfy their relevant criteria regardless of values for the parameters and thresholds for the quadrants.

#### Predicting a stable counter with positive and negative channels

**Figure 6:**
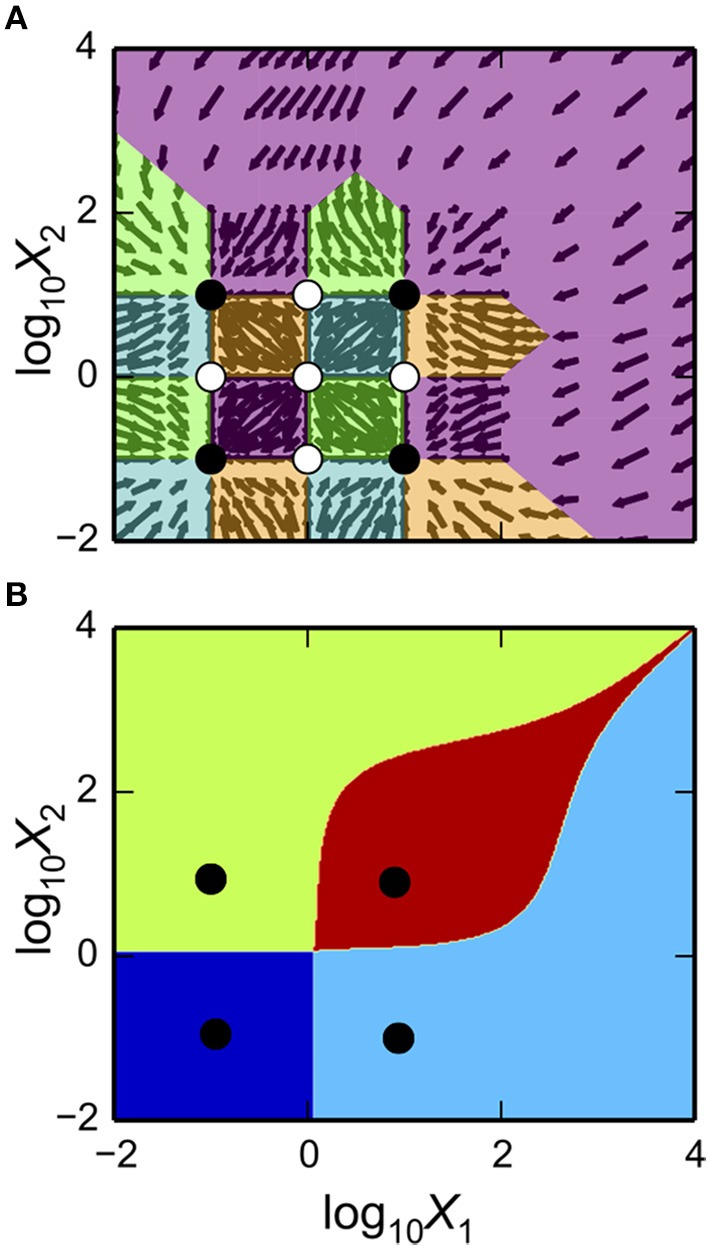
**Dynamic behavior of a quadrastable instance of the synthetic gene circuit. (A,B)** The x-axis represents the logarithm of the concentration of the first activator, X_1_; the y-axis represents the logarithm of the concentration of the second activator, X_2_. **(A)** State-space deconstruction of the gene circuit by system design space showing qualitatively-distinct trajectories. The steady states of the system are represented by black circles (stable) and white circles (unstable). The colors of the different regions correspond to regions with different qualitatively-distinct trajectories as described in the caption of Figure 5. **(B)** The basin of attraction, represented by the colored regions, represent the domains of state space that are attracted to a particular stable steady state (black circles). The boundaries between the basins of attraction are obtained by refinement using the original equations.

One arrangement of particular interest has one steady-state attractor that occupies each of the quadrants—consistent with four binary boolean states, represented by (−, −), (−, +), (+, −), and (+, +). We find that all of the ensembles identified in Section Predicting Parameter Sets for Realization of Multi-Stability are able to yield this particular arrangement of steady-state attractors, an example of which is shown in Figure 6, where *X*_*r*_,_1_ = 1 and *X*_*r*_,_2_ = 1.

This combination of (−, −), (+, −), (−, +), and (+, +) binary boolean states makes this design useful as a control switch where the expression of target genes are regulated by *X*_1_, *X*_2_ or both. For example, this synthetic circuit, controlling a reporter gene whose synthesis is directly coupled to *X*_1_ and inversely coupled to *X*_2_, can effectively count from 0 to 3 at well-defined levels for its expression. Such a reporter under the control of this module is modeled mathematically by the following ODE

(31) $$\begin{array}{r} \frac{dX_{4}}{dt}=\alpha_{4}\left(\frac{\rho_{41}X_{1}^{2}+K_{1}^{2}}{X_{1}^{2}+K_{1}^{2}}\right)\left(\frac{\rho_{42}^{-1}X_{2}^{2}+K_{2}^{2}}{X_{2}^{2}+K_{2}^{2}}\right)-\beta_{4}X_{4} \end{array}$$

where *X*_4_ represents concentration of the reporter protein; α_4_ represents the rate of synthesis of *X*_4_ at an unrepressed and inactivated state; β_1_ represents the rate constant for loss of *X*_4_ by dilution due to exponential growth; ρ_41_ represents the capacity for activation of *X*_4_ synthesis by *X*_1_; ρ_42_ represents the capacity for repression of *X*_4_ synthesis by *X*_2_.

The ability of this design to perform as a stable counter arises from the *X*_1_–*X*_2_ heterodimer formation in combination with the seemingly independent positive feedback loops for *X*_1_ and *X*_2_. For example, a transient increase in one species elicits a transient drop in the other that, in combination with the positive feedback loops, can lead to a switch from a stable “+” state to a stable “−” state.

**Figure 7:**
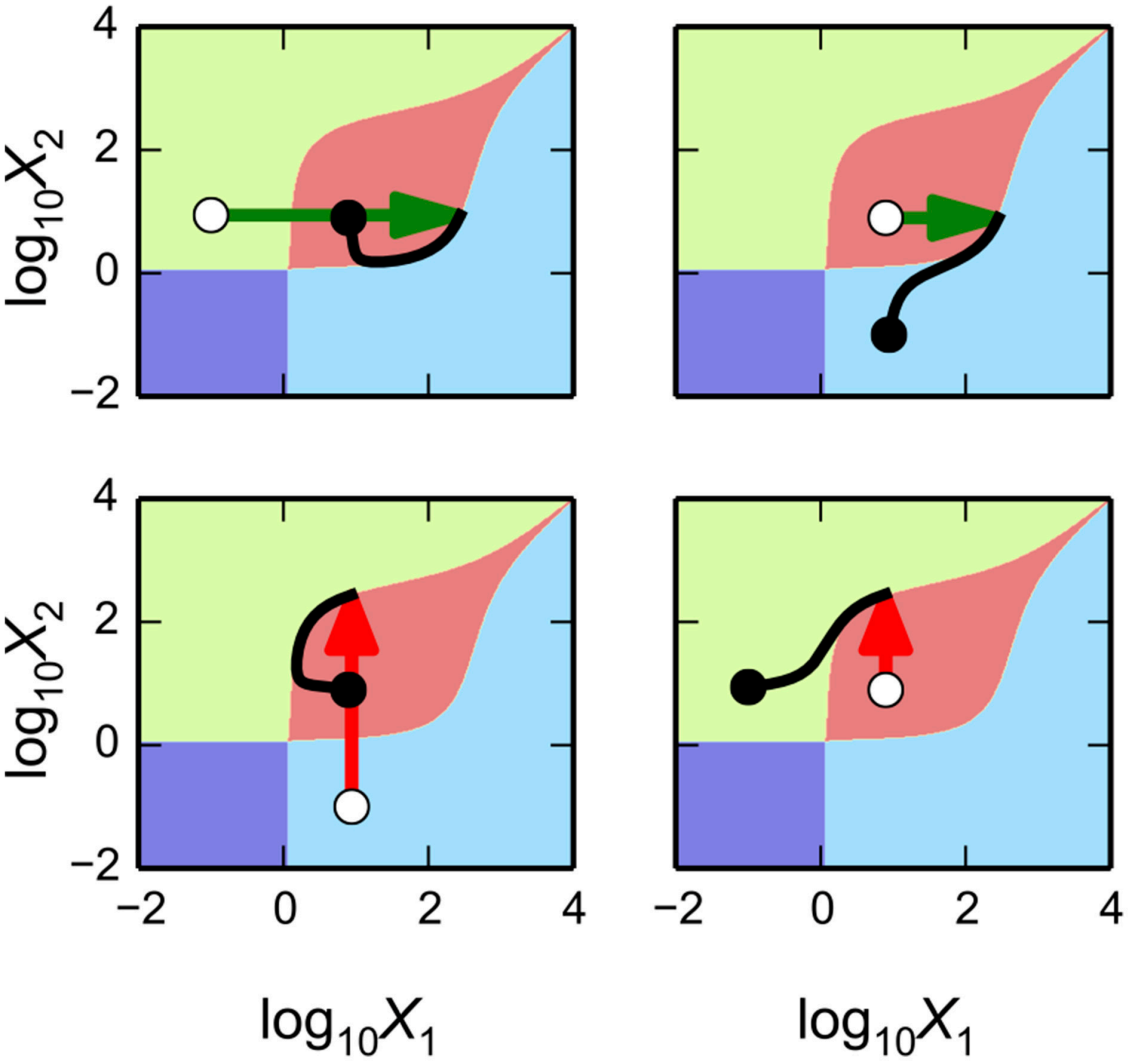
**Basins of attraction for a 4-state genetic counter**. The x-axis represents the logarithm of the concentration of the first activator, X_1_; the y-axis represents the logarithm of the concentration of the second activator, X_2_. Different colored regions represent values for the activators that converge to a unique steady-state attractor. Transitions from an initial steady state (white circle) to a new steady state (black circle) following an equal size bolus (275 μM) in one of the two activators. The top panels show transient simulations following a bolus of X_1_ (green arrows). The bottom panels show transient simulations following a bolus of X_2_ (red arrows). Left and right sub-panels show the transitions from different initial steady-state attractors.

This is reflected in the teardrop-shaped basin of attraction for the steady-state attractor in the (+, +) quadrant: when the system is at the (+, +) attractor and there is a transient increase in the concentration of either *X*_1_ or *X*_2_, the dynamics of the system are such that it leaves the basin of attraction for the (+, +) attractor and enters the basin of attraction for the (+, −) or (−, +) attractor, respectively. A visual representation of the transitions between the steady-state attractors following transient stimulation is shown in Figure 7.

Assume that the system is poised at the attractor in the (−, +) quadrant; if *X*_1_ is added in some amount, i.e., 275 μM, the system transitions to the attractor in the (+, +) quadrant; then if *X*_1_ is added again in the same amount, a transition to the attractor in the (+, −) quadrant ensues; therefore, by adding the same bolus of *X*_1_ twice, in a step-wise fashion, the system has switched between an equal number of steps, which bears the signature of a genetic counter.

Now, assume the system is poised at the opposite attractor in the (+, −) quadrant; if *X*_2_ is added in the same amount the system transitions to the attractor in the (+, +) quadrant; then if *X*_2_ is added again in the same amount, a transition to the attractor in the (+, −) quadrant ensues; therefore, by adding the same bolus of *X*_2_ twice, in a step-wise fashion, the system has reverted back to the original state.

**Figure 8:**
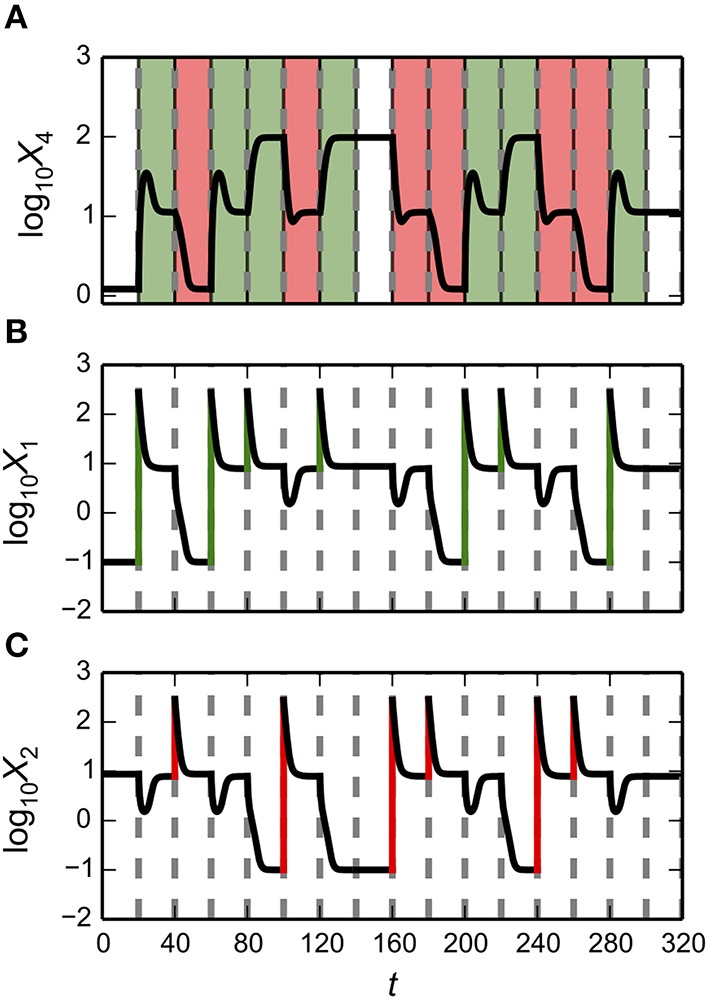
**Simulation of the counter following stimulation of the positive and negative channels**. Simulation of system behavior following a series of transient stimulations at regular intervals of 20 time units (dashed vertical lines). **(A)** Lines represent the concentrations of the reporter corresponding to the counter *X*_4_; **(B)** the positive channel *X*_1_; and **(C)** the negative channel *X*_2_. Transient stimulation of the positive channel, green vertical lines in **(B)**, results in an increase in the counter state, green background in **(A)**. Transient stimulation of the negative channel, red vertical lines in **(C)**, results in a decrease in the counter, red background in **(A)**. Time intervals without stimulation through either channel show that the count is stable, as shown by the white background in **(A)**.

These traits show that the system has two distinct channels that enable two sequences of transitions between the same three states but in the opposite order. A positive channel for (−, +) → (+, +) → (+, −) and a negative channel for (+, −) → (+, +) → (−, +). By coupling the module with the reporter gene, we show that the system is capable of counting between three levels of reporter concentration and can perform basic arithmetic using values 0, 1, and 2. An example showing a sequence of additions and subtractions following transient addition of *X*_1_ and *X*_2_, respectively, is shown in Figure 8.

## Conclusions

The Design Space Toolbox V2 is a compendium of tools designed to aid in the analysis and design of biochemical systems. It is particularly useful for the characterization of system design principles. Indeed, each of the “landmarks” in system design space—boundaries and vertices—are rigorously defined by particular constellations of parameter values that represent the “design principles” of the system (e.g., Savageau and Fasani, 2009). These constellations are not at all obvious and would be difficult to discover by trial and error, but are automatically determined with our tools. As in other engineering disciplines, knowing such design principles allows one to control the system in a more rational fashion.

These tools have already proven useful for understanding complex natural circuitry (Savageau, 2013) and for rationally designing and engineering new synthetic gene circuits (Lomnitz and Savageau, 2013, 2014, 2015) described by models composed of power functions from chemical kinetics and rational functions from biochemical kinetics. However, the full scope of models that can be analyzed by these new tools has yet to be explored.

These tools automate the construction and analysis of the design space of biochemical systems in a manner similar to a previous iteration of software tools known as the design space toolbox for Matlab®. However, this new iteration is a complete redesign of the approach that expands the scope of applicable systems beyond what was previously possible due to limits on both time and computational resources. The most important contribution provided by these tools is the enabling of a radically new phenotype-centric modeling strategy (Lomnitz and Savageau, 2015) that inverts the steps in the conventional parameter-centric strategy and automates those that are most difficult.

To illustrate our software tools, we applied them to the design of a synthetic two-gene circuit that has positive feedback loops with the potential for hysteretic-switch behavior. However, unlike other hysteretic switch designs that exhibit typical bistability (e.g., Gardner et al., 2000; Atkinson et al., 2003), this circuit has two seemingly independent positive feedback loops that are coupled by a fused heterodimerization domain. In an automated analysis, we show that this design can be tuned to exhibit up to four stable steady states. Furthermore, our tools predict multiple sets of values for the parameters that realize specific instances of the system that exhibit bistability, tristability and quadrastability.

Further analysis of a quadrastable instance of the system reveals that it can alternate between three of the steady states following transient stimulation in one of two input channels: a positive channel that results in the forward transition between these states; and a negative channel that results in the reverse transition between these same states. By coupling this network to a reporter gene, we have shown that this circuit can effectively count between three levels of fluorescence intensity in a step-wise manner.

These examples show the power of our new tools and illustrate how they enable a radically new modeling strategy that does not rely on first establishing nominal values for the parameters. Instead, this phenotype-centric strategy enumerates the phenotypic repertoire, identifies phenotypes of interest according to specific criteria, and then predicts sets of parameter values for realizing the phenotypes of interest. By assembling a variety of criteria, these tools can predict instances of a system that displays a rich assortment of behaviors.

## Author contributions

MS conceived the initial approach; JL and MS developed the methodology and wrote the manuscript; JL developed the software.

## Funding

This work was supported in part by a grant to MAS from the US Public Health Service, National Institutes of Health (RO1-GM30054).

### Conflict of interest statement

The authors declare that the research was conducted in the absence of any commercial or financial relationships that could be construed as a potential conflict of interest.
